# Mitochondrial Uncoupling Proteins: Subtle Regulators of Cellular Redox SignalingReviewing Editors: *Jerzy Beltowski, Joseph Burgoyne, Gabor Csanyi, Sergey Dikalov, Frank Krause, Anibal Vercesi, and Jeremy Ward*


**DOI:** 10.1089/ars.2017.7225

**Published:** 2018-09-01

**Authors:** Petr Ježek, Blanka Holendová, Keith D. Garlid, Martin Jabůrek

**Affiliations:** ^1^ Department of Mitochondrial Physiology, Institute of Physiology of the Czech Academy of Sciences, Prague, Czech Republic.; ^2^ UCLA Cardiovascular Research Laboratory, David Geffen School of Medicine at UCLA, Los Angeles, California.

**Keywords:** mitochondrial uncoupling proteins, UCP2, fatty acid cycling, attenuation of superoxide formation, redox signaling, anion transport

## Abstract

***Significance:*** Mitochondria are the energetic, metabolic, redox, and information signaling centers of the cell. Substrate pressure, mitochondrial network dynamics, and cristae morphology state are integrated by the protonmotive force Δ*p* or its potential component, Δ*Ψ*, which are attenuated by proton backflux into the matrix, termed uncoupling. The mitochondrial uncoupling proteins (UCP1–5) play an eminent role in the regulation of each of the mentioned aspects, being involved in numerous physiological events including redox signaling.

***Recent Advances:*** UCP2 structure, including purine nucleotide and fatty acid (FA) binding sites, strongly support the FA cycling mechanism: UCP2 expels FA anions, whereas uncoupling is achieved by the membrane backflux of protonated FA. Nascent FAs, cleaved by phospholipases, are preferential. The resulting Δ*p* dissipation decreases superoxide formation dependent on Δ*p*. UCP-mediated antioxidant protection and its impairment are expected to play a major role in cell physiology and pathology. Moreover, UCP2-mediated aspartate, oxaloacetate, and malate antiport with phosphate is expected to alter metabolism of cancer cells.

***Critical Issues:*** A wide range of UCP antioxidant effects and participations in redox signaling have been reported; however, mechanisms of UCP activation are still debated. Switching off/on the UCP2 protonophoretic function might serve as redox signaling either by employing/releasing the extra capacity of cell antioxidant systems or by directly increasing/decreasing mitochondrial superoxide sources. Rapid UCP2 degradation, FA levels, elevation of purine nucleotides, decreased Mg^2+^, or increased pyruvate accumulation may initiate UCP-mediated redox signaling.

***Future Directions:*** Issues such as UCP2 participation in glucose sensing, neuronal (synaptic) function, and immune cell activation should be elucidated. *Antioxid. Redox Signal.* 29, 667–714.

**Table:** 

Table of Contents	
I. Introduction	668
II. The Family of Mitochondrial UCPs	669
A. UCPs belong to the standard oxidative phosphorylation machinery	669
1. The SLC25 family of mitochondrial anion carrier proteins	669
2. Mitochondrial UCP subfamily	669
B. How much do UCPs uncouple?	669
III. The Mechanism of Uncoupling by the UCPs	670
A. Overview	670
B. The FA cycling mechanism: UCP as an FA anion flippase	670
C. The FA shuttling–carrier mechanism	671
D. Nascent FA requirement for UCP function and synergy with mitochondrial phospholipases	672
IV. Structure of Mitochondrial UCPs	673
A. Structure of UCP2	673
1. The structure of SLC25 family of mitochondrial anion carrier proteins	673
2. The detailed structure of UCP2	674
B. Structure of FA binding and anion binding site	674
C. Structure of nucleotide binding site	675
V. Regulation of *UCP* Gene Expression	676
A. *Ucp2* and *ucp3* genes	676
B. UCP2 transcription	676
C. Regulation of UCP2 translation	678
D. Turnover of UCP2	678
E. Post-translational modifications of UCP2	678
F. Regulation of UCP3 expression	678
G. Regulation of UCP4 and UCP5 expression	679
VI. Redox Homeostasis and Mitochondrial and Cell Redox Regulations	679
A. Mitochondrial redox state frequently regulates cellular redox state	679
1. Distinct nature of mitochondrial ROS sources	679
2. Uncoupling as a mechanism downregulating mitochondrial superoxide formation	683
3. Attenuation of superoxide formation by UCPs	683
B. Mitochondrion as major hub for cell redox signaling	685
C. Hypothetical assumptions for UCP participation in redox signaling	686
VII. Noncanonical Roles of Mitochondrial UCPs	687
A. Extrusion of organic anions from the matrix by UCP2-mediated antiport	687
B. Mutual relationships between the FA cycling and the anion transport function	688
C. Relationships between uncoupling and mitochondrial calcium transport	688
D. Involvement of UCPs in mitochondrial network dynamics and cristae morphology	689
1. Mild uncoupling promotes fission and mitophagy	689
2. Mild uncoupling reshapes cristae	690
VIII. Involvement of UCPs in Redox Homeostasis and Redox Regulations	690
A. Regulation of redox-sensitive kinase signaling by UCPs	690
B. Regulation of insulin secretion	691
C. Redox regulations in endothelial cells	692
D. Redox regulations of cell cycle	692
E. UCP involvement in the central regulation of metabolism	693
F. UCP involvement in cardioprotection	693
G. UCP involvement in brain and neuroprotection	694
H. UCP involvement in cancerogenesis	695
I. Involvement of UCPs in immune cells	695
IX. Future Prospects	698

## I. Introduction

Mitochondrial uncoupling proteins (UCPs), except for the brown adipose tissue UCP1, are reviewed here with emphasis on their effects on reactive oxygen species (ROS) homeostasis and concomitant redox regulations. Redox regulations arise from sudden and often transient shifts in the redox homeostasis in a certain closed compartment. Their main characteristic is the ability to spread, in our case from mitochondrion to the cytosolic and even to the extracellular environment *(retrograde signaling)* or *vice versa* (*cell signaling to mitochondrion*). This property is projected to the important physiological regulatory functions of UCPs, as based on the ability of mitochondrial UCPs to attenuate mitochondrial superoxide formation (but not for all mitochondrial sources such as those arising from mtDNA mutations).

First, we provide an overview of the family of mitochondrial UCPs. We present the current understanding of the basic mechanism of UCP-mediated uncoupling and the structural bases for its transport mechanism and regulation. *UCP* genes and the regulation of their expression are discussed.

We then proceed to illustrate the involvement of UCPs in redox homeostasis and predict hypothetical rules for direct or indirect UCP participation in redox signaling. We discuss conditions that can be affected by mild uncoupling and those that cannot be influenced, and finally, those in which UCPs are physiologically switched on/off. We also put into context the newly revealed ability of UCP2 to expel aspartate, oxaloacetate, and malate from the matrix in exchange with phosphate. A synthesis of these aspects provides predictions for UCP roles in various physiological phenomena. We compare these predictions with reported findings and propose a universal view of UCP physiology.

We strictly distinguish between mitochondrial compartments into which superoxide is released from sources, typically residing within the inner mitochondrial membrane (IMM). Superoxide can be released into the mitochondrial matrix or to the intracristal space (ICS) due to the existence of rich enfolded cristae formed by IMM (329). Only a minor superoxide release into the external intermembrane space takes place. The intermembrane space represents only a thin compartment within the sandwich of the cylindrical outer mitochondrial membrane (OMM), forming tubules of mitochondrial reticulum and the inner boundary membrane (bottom sandwich part formed by the cylindrical IMM portion).

To stay within the scope of this review, we leave out the topic of the role of UCP1 in thermogenesis, in obesity, in adipose tissue development, and preadipocyte differentiation. The reader can refer to excellent reviews on these subjects in (72, 185, 217). Also beyond the scope of this review are the role of constitutively expressed UCP1 in thymocytes as a factor in determining T cell population selection (4, 76) and mitochondrial UCPs in plants (410) and unicellular eukaryotes (432).

## II. The Family of Mitochondrial UCPs

### A. UCPs belong to the standard oxidative phosphorylation machinery

#### 1. The SLC25 family of mitochondrial anion carrier proteins

The SLC25 anion carrier gene family involves specifically mitochondrial carriers or channels residing as the integral membrane proteins within the IMM. Predominantly, these carriers ensure anionic substrate traffic into or from the mitochondrion. Despite the fact that the family carriers possess a common structural organization with six transmembrane α-helices and a specific sequence signature, they ensure different transport modes for numerous organic anions—from an electrophoretic ADP^2−^/ATP^3−^ antiport, *via* the electroneutral oxoglutarate^2−^/malate^2−^ antiport, or phosphate*H^+^ symport up to the uniport of hydrophobic anions such as fatty acids (FAs) by UCPs (201, 316).

#### 2. Mitochondrial UCP subfamily

Decades of studies have brought a clear picture of molecular function as well as physiological relevance of mitochondrial UCPs. This competitive field has been typically accompanied by incompatible mutually exclusive hypotheses on molecular mechanism of uncoupling and by distinct views of their physiological roles (20, 210, 226). Progress in structural and molecular biology and detailed cell and systemic studies provided convergence in the previously divergent field.

**FIG. 1.**
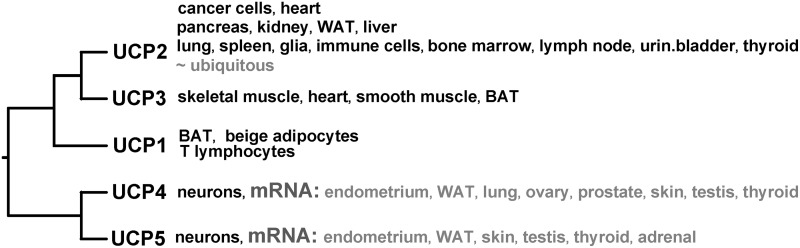
**Human UCP isoforms and their tissue distribution.** Phylogenetic tree of human UCPs based on their primary amino acid sequence, depicted together with their major tissue/cell distribution; sequences of human UCPs were aligned using ClustalW 2.0 and displayed as rooted phylogenetic tree. *Black*: existence of protein verified; *gray*: only mRNA detected. BAT, brown adipose tissue; UCP, uncoupling protein; WAT, white adipose tissue.

The classic uncoupling protein UCP1 has long been known and recognized for its thermogenic function, exclusively in the special bioenergetics setup of oxidative phosphorylation (OXPHOS) machinery in brown adipose tissue mitochondria (226). Besides UCP1, the initial skepticism for function of other UCP isoforms (Fig. 1), discovered in 1996–1999, originated from their minute amounts specifically variable in distinct tissues. UCPs form a distinct clad in the phylogenic tree of the SLC25 anion carrier gene family (153, 184, 201, 209, 227, 316).

A complete tissue pattern of UCP isoforms is not always known or is controversial for specific tissues or cell types, even for tissues such as heart or pancreas, including pancreatic β-cells. For example, UCP4 protein has been found preferentially in neurons (379, 380), but numerous articles refer to UCP2 in neuronal mitochondria (see sections VIII.E and VIII.G). An ingenious concept of mild uncoupling serving for attenuation of mitochondrial superoxide formation has sparked new avenues for discovering unexpected roles for UCPs, involving a role in decision-making for immune cells, apoptosis regulation, regulatory role for insulin secretion, *etc*.

### B. How much do UCPs uncouple?

OXPHOS at mitochondrial ATP-synthase (complex V) is driven by the protonmotive force, Δ*p* (Δ*p* = Δ*Ψ* + Δ*pH*, in mV), formed by the respiratory chain H^+^ pumping at complexes I, III, and IV. The F_O_ATPase (1), that is, the IMM domain of ATP-synthase, consumes an adequate Δ*p* portion in a state, historically termed state-3 for isolated mitochondria (210).

*In vivo* where cellular respiration is governed by the metabolic state and/or availability of substrates, a finely tuned spectrum of various “states-3” can be established, depending on the substrate load (*e.g.*, increasing glucose). A state-4 is then given by zero ATP synthesis in isolated mitochondria, when zero H^+^ backflux *via* the F_O_ATPase proceeds while respiration and H^+^ pumping proceed due to the so-called H^+^ leak, mediated nonspecifically by surfaces of integral membrane proteins or by the native H^+^ permeability of the IMM lipid bilayer, plus the regulated H^+^ backflux mediated specifically by active UCPs. Since mitochondrial Δ*p* exists predominantly in the form of Δ*Ψ*_m_, IMM electrical potential, the highest Δ*Ψ*_m_ is established at state-4 and the maximum substrate load.

Common attributes lie in the ability of UCPs, when anion cycling substrates are accessible (see section III), to prevent superoxide burst in a particular moment of peaking redox regulations, or else, to enhance or, hypothetically, to initiate the increase of superoxide formation, and consequently of downstream mitochondrial oxidants, when UCP function is suppressed. The key disputes during the years of research have thus concerned the issue of how UCPs are activated, which is also reviewed hereunder.

But the main questions still remain: (i) how much the protonmotive force Δ*p* has to be diminished to yield a significant attenuation of mitochondrial superoxide formation? (ii) Do UCPs other than UCP1 possess the ability to dissipate Δ*p* below such threshold magnitudes?

Current assessment stems from the fact that the content of UCP2, UCP3, UCP4, and UCP5 in tissues is so low that under basal conditions, that is, with low endogenous nascent FA levels, UCPs do not uncouple by a strength that would disable OXPHOS. This means that no significant decrease of Δ*p* is induced as established on the IMM with the two components Δ*Ψ*_m_ and Δ*pH*. Such a basal UCP contribution can be added to the well-described concept of the IMM proton leak (20). In contrast, when the mild uncoupling is switched on, the magnitudes of Δ*p* (Δ*Ψ*_m_) decrease stay within the range of several millivolts and do not fall below the threshold when OXPHOS is stopped (such as at artificial uncoupling with chemical uncouplers, for example, carbonyl cyanide 4-(trifluoromethoxy)phenylhydrazone [FCCP]).

The range of UCP1 uncoupling in brown fat mitochondria encompasses ∼55 mV but estimates for UCP2 in lung gave maximum of 12.5 mV (210, 296). Maximum protonophoric activity of UCP1 has been evaluated as 20 μmol min^−1^ *per* mg UCP (136); that is, 333 nmol s^−1^ *per* mg UCP, which is equal to number of turnovers of 11 s^−1^. Reconstitution and planar lipid bilayer membrane (BLM) electrophysiology experiments show a similar maximum rate *V*_max_ for UCP2 as for UCP1 (29, 179, 182). Consequently, the extent of Δ*Ψ*_m_ drop caused by UCP2 will be given by the expressed UCP2 protein amount in the given tissues, exactly by a fraction of activated UCP2 molecules. Since the UCP2 amounts are two orders of magnitude lower, resulting uncoupling cannot exceed 10 to 15 mV. For example, the level of UCP2 in spleen mitochondria is <1% of the level of UCP1 in brown adipose tissue mitochondria (320).

## III. The Mechanism of Uncoupling by the UCPs

### A. Overview

The UCPs exhibit two transport modalities: in the presence of FAs, they catalyze electrophoretic transport of protons (H^+^ uniport), which is the cause of mitochondrial uncoupling and energy dissipation. They also catalyze electrophoretic transport of selective anions, notably including Cl^−^ (UCP1) and hydrophobic anions such as alkylsulfonates (205).

The role of FA in UCP-mediated uncoupling was the subject of much debate. Early leaders of the field maintained that UCP1 catalyzed H^+^ transport. As stated emphatically by Nicholls: “Thus it was clear by 1974 that UCP1 could conduct protons in the strict absence of fatty acids, eliminating the possibility that fatty acids play an obligatory cycling role in the mechanism of proton translocation by UCP1” (297).

This statement, however, was based on studies carried out on isolated mitochondria. Such studies are not reliable, because FAs are continuously produced by the action of phospholipases acting on membrane phospholipids (124). When confronted by a problem of this nature, it is necessary to follow the advice of Palmieri: purification and reconstitution in artificial membranes are essential for a detailed functional characterization of a transport protein (315). Use of these techniques has firmly established that FAs are obligatory for the activity of UCPs. This has been demonstrated in proteoliposomes (31, 138, 179, 181, 182, 205, 431, 444), in black lipid membranes (28, 29, 356, 406), and in patch clamp studies of the IMM of brown adipose tissue mitochondria (124).

These results place severe constraints on the UCP transport mechanism: it must explain transport of both protons and anions, and it must also explain the role of FA in UCP-mediated uncoupling. Two models meet these criteria.

### B. The FA cycling mechanism: UCP as an FA anion flippase

**FIG. 2.**
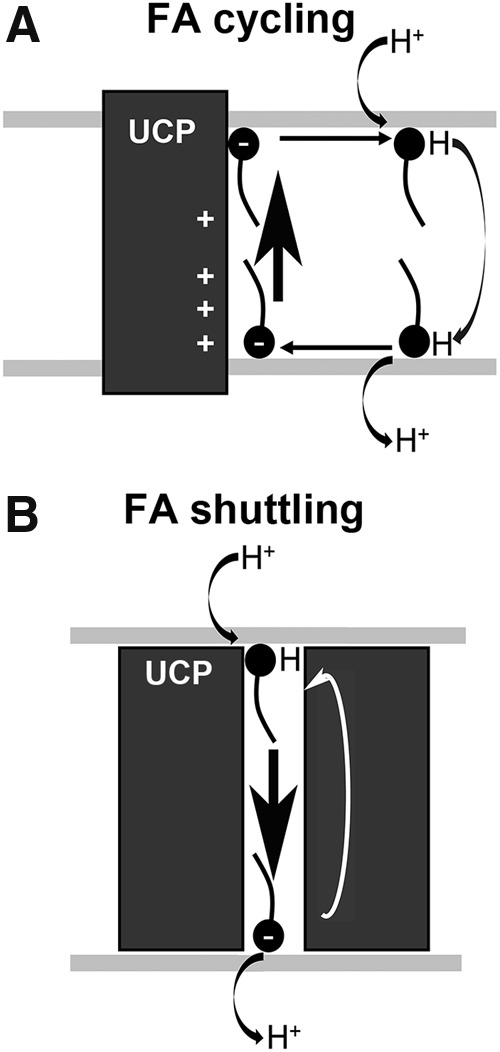
**The UCP-catalyzed protonophoretic cycle—ongoing according to the (A) FA cycling or (B) “FA shuttling.” (A)** In FA cycling model, FA^−^ anion diffuses laterally within the membrane to reach a subsurface peripheral UCP binding site near the matrix (196), where it binds specifically to basic residues Arg60 and Lys271 (depicted as **+**) (31). The IMM potential drives the carboxylate head group through an electrostatic path composed of basic residues both inside and outside the UCP cavity (31), resulting in a transport of FA^−^ anion to the other side of the membrane (*vertical arrow*), that is, to the ICS-proximal lipid leaflet of ICS membranes (parts of IMM enfolded into cristae). The anion diffuses laterally (*horizontal arrows*) away from UCP, where it is protonated. Protonated FA diffuses rapidly back across the membrane to deliver protons electroneutrally back to the matrix by a spontaneous flip-flop mechanism, completing the cycle (182). **(B)** “FA shuttling” mechanism, in fact considering protein as a “carrier” where FA shuttles back and forth (wobbling) (124), actually differs so that the FA molecule cannot diffuse away from the UCP protein and stays in an unspecified way bound to the protein while exposed to the *cis* or *trans* side of the membrane either as anion or after protonation. In this case, both anionic and neutral protonated FAs are carried through the UCP protein. However, since the actual binding site was verified to face the lipid bilayer (31), this mechanism is very unlikely. FA, fatty acid; ICS, intracristal space; IMM, inner mitochondrial membrane.

UCP-mediated anion transport is the key to understanding the uncoupling mechanism, because the inorganic anion uniport plays no physiological role—a “case of the dog that didn't bark.” The first major advance was confirmation that UCP catalyzes guanosine-diphosphate (GDP)-sensitive halide transport (196, 204) and that UCP-mediated Cl^−^ transport is inhibited by FA (205). This was observed both in brown adipose tissue mitochondria and in proteoliposomes reconstituted with UCP1. These results led to the hypothesis that UCP is an anion channel designed to conduct FA anions and does not transport protons. According to this mechanism, the function of UCP is to permit FAs, whose anions are normally unable to cross biomembranes, to act as cycling protonophores (Fig. 2A). A similar mechanism was proposed by Skulachev (373).

In support of the hypothesis, only FAs capable of spontaneous diffusion across the phospholipid membrane by a flip-flop mechanism (218–220) are able to induce UCP-mediated H^+^ translocation (183, 202, 203). Moreover, FA molecules must be unipolar. Dicarboxylic FAs or FAs with bulky groups in the ω-end do not exhibit UCP uncoupling (48, 202, 203). Thus, the presence of UCP in biomembranes is not sufficient for uncoupling; also required are FAs able to flip-flop across the membrane and to be protonated and deprotonated.

The second major advance was finding a wide variety of monovalent anions that are transported by UCP and competitively inhibit UCP-mediated Cl^−^ transport (191,196). Among them, the long-chain alkylsulfonates stand out, because they are FA analogues. They are transported electrophoretically by UCP, but they cannot induce uncoupling, because their pKa <1, and they cannot be protonated for the flip-flop part of the cycle (134, 135, 138, 183, 202). This limitation can be overcome by ion pair transport of alkylsulfonates with amphiphilic bases, such as propranolol (137). Indeed, full GDP-sensitive uncoupling in proteoliposomes and brown adipose tissue mitochondria is observed in the presence of propranolol and undecanesulfonate (183). This supports the hypothesis that the mechanism of uncoupling requires outward transport of the anionic head group by UCPs and that the H^*+*^ transport occurs *via* the bilayer and not *via* UCP (134, 135, 138, 183, 193, 205).

A comparative study of H^+^ and anion fluxes induced by laurate and its close analogue, undecanesulfonate (C11), yielded the following results: the analogues have very similar *K*_m_ values, they inhibited each other competitively and they both inhibited Cl^−^ transport competitively. Fluxes induced by laurate and C11 were inhibited by GDP. The only difference is that laurate caused UCP-mediated H^+^ transport, whereas C11 did not. Given the simple fact that C11 is transported by UCP, there is no physicochemical basis for excluding FA anion transport by UCP. On these grounds, it has been concluded that the physiological function of UCP is to catalyze electrophoretic efflux of FA^−^ anions from the matrix, leading to futile cycling of FA (Fig. 2A) (135, 136, 138). Importantly, the same conclusion applies to uncoupling by UCP2 and UCP3 (29, 31, 179, 181, 182, 194, 208, 210, 268, 444).

An important mutagenesis and functional study of UCP2 by Berardi and Chou (31) provides strong support for the FA cycling hypothesis. They found that ionized FA head group binds specifically to a matrix site of UCP2 *via* electrostatic interaction. The ionized head group is transported through an electrostatic path constituting basic amino acid residues both inside and outside the UCP2 cavity (31). In addition, FA binding to UCP2 and FA transport by UCP2 are tightly coupled to H^+^ translocation. This strongly supports the model in which external H^+^ (from the ICS) crosses the inner membrane as protonated FA and UCP2 subsequently allow the recycling of ionized FA.

The FA cycling mechanism has been engineered into synthetic “UCP mimics” (433). These synthetic compounds are able to perform FA-activated H^+^ translocation in phospholipid bilayers (433). Such hydrogen and halogen bond-based synthetic anion transporters possess poor H^+^/OH^−^ transport activity without FAs, but in the presence of long-chain FAs, they are switched on as proton transporters with an activity close to that of a commonly used protonophore, thus allowing the FA to complete a proton transport cycle. These studies provide an important proof of concept for the FA cycling hypothesis.

### C. The FA shuttling–carrier mechanism

Fedorenko *et al.* (124) studied UCP1 using patch clamp of the inner membrane of brown adipose tissue mitochondria. They reproduced earlier findings showing that UCP does not conduct protons in the absence of FA and that UCP catalyzes electrophoretic transport of alkylsulfonates (138, 183). Their results led them to introduce an alternative mechanism, called the FA-shuttling model (Fig. 2B). In this model, the FAs do not diffuse away from UCP to the phospholipid bilayer, but remain bound to the UCP by hydrophobic interactions. The protonated FA carries (shuttles) H^+^ to the matrix, not by spontaneous diffusion across the phospholipid bilayer, but through the protein. This is one of the major differences between the shuttling and cycling—in cycling, FA^−^ anion is translocated species, whereas in shuttling, both protonated and anionic FA^−^ move back and forth, respectively.

At the matrix, the FA is ionized and ejected through the same channel. In short, the FA “shuttles” back and forth through the transport channel. This highly unusual model can be disputed on theoretical grounds, and it is disproven by published experiments:

(i) Long-chain FAs remain bound to the UCP by hydrophobic interactions. They “cannot dissociate from UCP1” and “serve as permanently attached UCP1 substrates that help to carry H^+^ through UCP1.” These assertions require that the hydrophobic interaction of FA with the protein is much stronger than the hydrophobic interaction of FA with the bilayer. On theoretical grounds, this is highly unlikely.
(ii) UCP can only mediate inward H^+^ transport, that is, the uncoupling mechanism is asymmetric. Fedorenko *et al.* (124) attempted to establish a transmembrane gradient of FA anions by adding cyclodextrin to the pipette solution to deplete FA from the matrix membrane leaflet and 1 μ*M* oleic acid (OleA) to the bath solution to saturate the cytosolic leaflet with OleA anions. Under these conditions, the reversal potential should be negative if UCP1 transports FA^−^ anion from the matrix, and the current amplitude should be larger in the outward direction. However, the reversal potential was ∼0 mV, which corresponds to the equilibrium potential for H^+^ but not for FA^−^ anions, and the current was of equal amplitude in both directions.
On this basis, they conclude that UCP1 does not operate in accordance with the FA cycling model. It is fair to say that the UCP mechanism would have been solved long ago if it were possible to create a gradient of FA anions independently of the pH and protonated FA gradients. However, this is not possible. The protonated OleA crosses the membrane very rapidly and comes into acid–base equilibrium, thereby producing the observed results. This experiment, therefore, says nothing about either model.
(iii) UCP can only mediate inward alkylsulfonate transport, that is, UCP-mediated anion transport is asymmetric. Fedorenko *et al.* (124) compared currents when alkylsulfonates were added to the pipette solution (matrix side of UCP) with those obtained when added to the bath solution (cytosolic side of UCP). The transient currents observed with C11 and octadecanesulfonate (C18) were highly asymmetric, strongly favoring transport from the bath (cytosolic side) to the pipette (matrix side).
On this basis, the authors concluded that the anionic headgroup of alkylsulfonates, and, by analogy, FA, could only bind to UCP on the cytosolic side. This conclusion fails to take into account the large experimental asymmetry that was imposed. C11 and (C18) will partition almost entirely into the membrane leaflet. If the volume of the pipette solution is 10 μL and the volume of the bath is 1.0 mL, the membrane concentrations of C11 and C18 on the bath side will be nearly 100-fold higher than on the pipette side. This may explain the observed asymmetry. However, it is not necessary to speculate on this issue. Three independent experiments demonstrate conclusively that C11 sulfonate undergoes transport from the matrix face of UCP1: UCP1 was reconstituted in proteoliposomes that typically exhibit 50:50 inward:outward orientation of UCP. There was robust C11 influx when GDP was present outside the vesicle [Fig. 4, Ref. (183)], a condition in which C11 is being transported from the matrix side of UCP.

Propranolol was used to transport C11 electroneutrally across the bilayer (183). Propranolol and C11 form hydrophobic ion pairs that readily equilibrate across the membrane. Valinomycin induced robust GDP-sensitive H^+^ efflux in proteoliposomes containing UCP1 due to cycling of C11 and C11*propranolol ion pair. Importantly, this flux was observed when GDP was present outside the vesicle [Fig. 8, Ref. (183)], a condition in which C11 is being transported from the matrix side of UCP. Similar results were obtained with C9 and C15 sulfonates, which are analogues of decanoic and palmitic acids. Propranolol was also used to transport C11 into the matrix of brown adipose tissue mitochondria to observe C11 transport from the matrix side.

The results of these experiments may be summarized thus: ion pair transport converts C11 sulfonate into a pseudo FA that supports GDP-sensitive uncoupling of brown adipose tissue (BAT) mitochondria. These experiments confirm that alkylsulfonates are readily transported outward by UCP1.

The FA shuttling–carrier mechanism is thus disproven by experiment. It is important to note, however, that the work of Federenko *et al.* (124) introduced an important new approach to the study of UCPs.

### D. Nascent FA requirement for UCP function and synergy with mitochondrial phospholipases

Recently, a second rule was revealed for UCP2 functional activation *in vivo*, based on the observation that only those FAs instantly cleaved by mitochondrial phospholipases, such as calcium-independent phospholipase A2γ (iPLA_2_γ), induce UCP2-mediated uncoupling in cells (186). This phenomenon has been previously indicated with UCP1 studied by direct patch clamp of IMM of brown adipose tissue (124). The consequent involvement of iPLA_2_γ and UCP2 in the antioxidant synergy and redox regulations of, for example, both glucose- and FA-induced insulin secretions (186) is described hereunder. We can remark that such a mechanism, previously unexplained, led in the past to skeptical views, questioning the uncoupling role of UCP2–UCP5 *in vivo*.

Accumulating evidence suggests that the availability of FAs to induce UCP-mediated H^+^ transport *in vivo* is provided by mitochondrial phospholipases. Patch clamp studies of UCP1 currents in the native IMM of brown adipose tissue mitochondria indicated a calcium-independent phospholipase regulation of the UCP1 activity (124). However, the identity of the phospholipase(s) involved in UCP1 activation remains to be established.

**FIG. 3.**
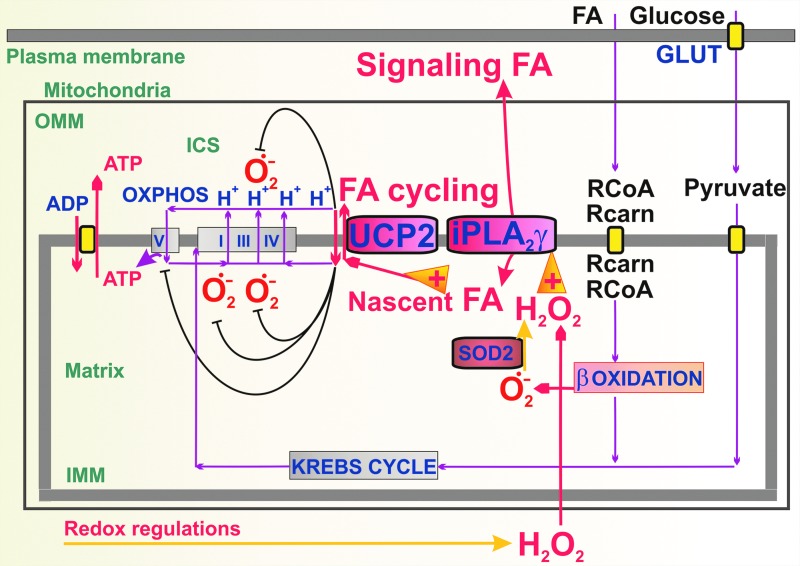
**Nascent FAs for UCP-mediated attenuation of superoxide formation are provided by the redox-activated mitochondrial phospholipase iPLA2γ**. The H_2_O_2_-activated mitochondrial phospholipase iPLA2γ (PNPLA8) has been identified in mitochondria that allows a direct feedback attenuation of mitochondrial superoxide production (180, 186). Upon redox activation that typically exists at β-oxidation of FAs (+ in the arrowhead), iPLA2γ cleaves IMM phospholipids and releases nascent free FAs, which become cycling substrates enabling UCP2-mediated uncoupling. The consequent partial dissipation of Δ*p* decreases mitochondrial superoxide formation. Moreover, FAs released by iPLA2γ serve as agonists for plasma membrane receptors such as GPR40 (186), hence FA signaling represents an amplified message. Δ*p*, protonmotive force; GPR40, G-protein-coupled receptor-40; iPLA_2_γ, calcium-independent phospholipase A2γ; OMM, outer mitochondrial membrane; OXPHOS, oxidative phosphorylation.

UCP2-dependent feedback downregulation of mitochondrial superoxide production has been characterized in detail. The addition of *tert*-butyl hydroperoxide or H_2_O_2_ to respiring mitochondria caused increase in respiration and decrease in membrane potential that was completely inhibited by bromoenol lactone, a selective inhibitor of calcium-independent phospholipases iPLA2 (180, 188). Because the peroxide-initiated uncoupling was also sensitive to carboxyatractyloside and purine di- and tri-phosphates, we concluded that it originated from the onset of FA cycling mediated by the ANT1 and UCP(s) (188). The following studies using mitochondria isolated from tissues rich in UCP2 (180) and insulinoma cells (186) identified redox-sensitive mitochondrial iPLA2γ as the main regulator of the UCP2 activity (Fig. 3).

Overall, the data are consistent with a mechanism of H_2_O_2_-activated iPLA2γ, with subsequent cleavage of phospholipids and release of free nascent FAs that are the cycling substrates of UCP2 and mediate uncoupling. The consequent partial dissipation of Δ*p* initiates a direct feedback attenuation of mitochondrial superoxide production (180, 186).

One may speculate that such a redox-initiated cleavage of mitochondrial phospholipids by iPLA2γ may also simultaneously increase the leakiness of the IMM to H^+^. However, this has not been observed. The absolute requirement of the iPLA2γ-dependent H^+^ transport either on ANT1 or UCP2 suggests that the iPLA2γ catalytic activity is under strict regulation. Studies using purified recombinant iPLA2γ reconstituted in phospholipid vesicles are consistent with reversible direct activation of the enzyme by H_2_O_2_ (186), suggesting a reversible oxidative modification of protein thiol(s).

## IV. Structure of Mitochondrial UCPs

### A. Structure of UCP2

#### 1. The structure of SLC25 family of mitochondrial anion carrier proteins

The subfamily of mitochondrial UCPs within the family of mitochondrial anion carrier proteins (SLC25) exhibits common structural features despite the diverse sequence identity. Thus, UCP1 protein shares 60% sequence identity with UCP2 and UCP3 and these two proteins share 70% of sequence identity with each other (39, 411). All mitochondrial carrier proteins developed from one common ancestor. The genes encoding for all mitochondrial carriers present a threefold duplication that results in a threefold repeat of about 100 amino acids within the proteins (316, 420). The carriers consist of six transmembrane helices H1–H6 with both amino and carboxy termini oriented toward the intermembrane space/ICS (16, 32, 201, 316, 420). The X-ray structure of the ADP/ATP carrier (or adenine nucleotide translocase, ANT) complexed with its inhibitor carboxyatractyloside was revealed at a resolution of 2.2 Å (319) in 2003.

The structure confirmed that the transmembrane domain consists of six α-helices, all of which are tilted relative to the orthogonal direction of the membrane and each other. The six helices form a barrel that defines a deep cone-shaped depression accessible from the outside (32, 319). The fold of the three repeats is very similar and the connections within pairs of odd and even numbered helices contain short α-helical stretches. Each of the odd-numbered helices exhibits a shark kink, which is due to the proline residue. These prolines are located in the conserved sequence PX(D/E)XX(K/R) characteristic of mitochondrial carriers (295).

Analysis of symmetry of different mitochondrial carrier family members revealed many interesting features regarding the structural and functional properties of UCP1 and other carriers (346). The charged residues within the PX(D/E)XX(K/R) sequence form a salt bridge network connecting the C-terminal end of the transmembrane helices, closing the transporter on the matrix side (32, 319). Analysis of the pseudosymmetry of UCP1 and other mitochondrial carriers revealed a highly conserved and symmetrical (FY)(DE)XX(RK) motif on the cytoplasmic site of the carrier, which also has the propensity to form a salt bridge network (346). In the cytoplasmic conformation state, the charged residues are not engaged in interactions, but they form a cytoplasmic salt bridge network when the carriers are in the matrix state (224, 357).

The residues of the cytoplasmic network are located on the even-numbered α-helices, whereas those of the matrix network are on the odd-numbered α-helices. Both networks are at the water–membrane interface on either side of the carrier, where solute access to the central substrate binding site may be controlled. In the carrier transport mechanism, substrate binding in one conformation allows the conversion to the other conformation by the disruption and formation of these networks, causing the alternating opening and closing of the carrier to either side of the membrane. In contrast, UCP1 has cytoplasmic/ICS network parts similar to that of the ADP/ATP carrier of yeast, consisting of two salt bridges and one hydrogen bond. The complete conservation of both salt bridge networks in UCP1 suggests that it also has an alternating access mechanism (83).

#### 2. The detailed structure of UCP2

**FIG. 4.**
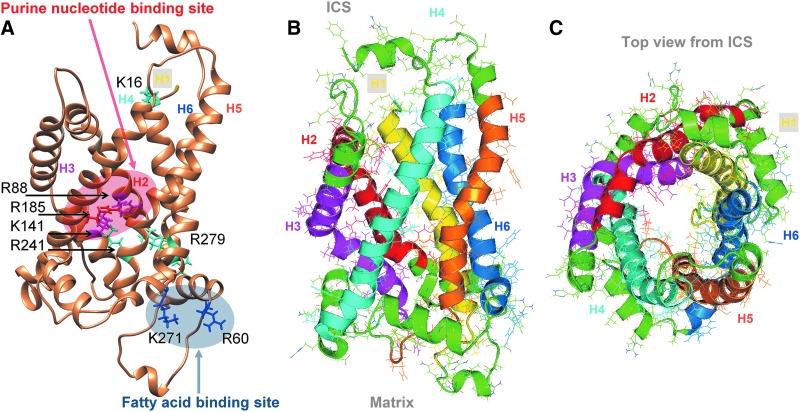
**Structure of UCP2**—**according to Refs. (31) and (32)**. **(A)** UCP2 with depicted basic amino acids responsible for FA transport and purine nucleotide binding. **(B)** Side view. **(C)** Top view from the “cytosolic” side, that is, from the ICS side. *H1–H6*: color-coded transmembrane α-helices. The peripheral basic residues Arg60 and Lys271 are responsible for the binding of the carboxylate head group. Peripheral Arg241 together with residues inside the cavity, including Lys16, Arg88, Lys141, and Arg279 contribute to the flipping of the acidic head group through the protein cavity. In addition, the cavity-lining basic residues Arg88, Lys141 together with Arg185 participate in purine nucleotide binding (31). The structure was derived from the published NMR structure of the mitochondrial UCP2, pdb code 2LCK (32), and processed using Swiss Pdb-Viewer v. 4.1.0 (146) and the PyMOL Molecular Graphics System Version 1.8 Schrodinger, LLC.

In 2011, the structure of UCP2 in complex with UDP was revealed by NMR using chemical shifts of backbone ^1^H^N^,^15^N, and ^13^C' nuclides, deriving orientation restraints from residual dipolar coupling (32) (Fig. 4). The overall conformation resembles one of the ADP/ATP carriers (319) despite their low (∼20%) sequence identity. The three repeats adopt similar folds and the UCP2 structure also has kinks at the proline residues conserved in the SLC25 carrier family. The most significant differences between UCP2 and ANT are located in the third repeat. In each of the three repeats of the ANT carrier, the amphipathic helix packs against the segment of the odd-numbered transmembrane helix that follows the conserved proline, and the proline kink in the transmembrane helix closes the channel.

**FIG. 5.**
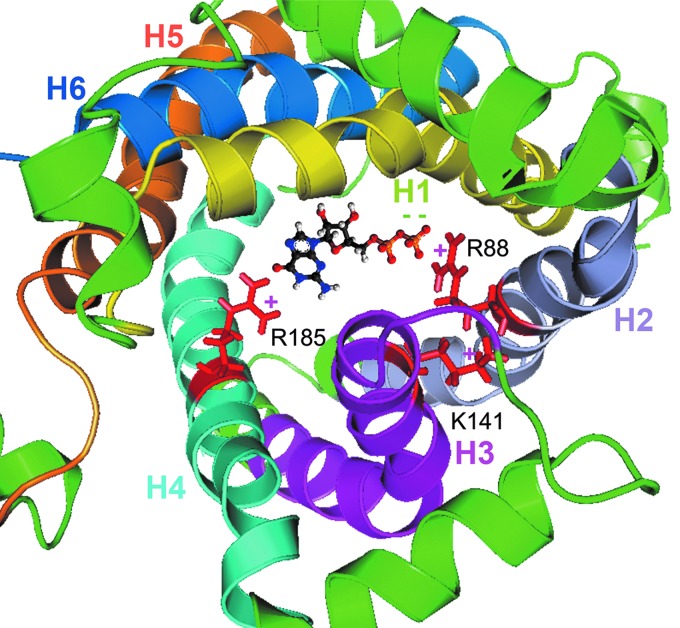
**Detailed structure of UCP2 purine nucleotide binding site**. Visualization of UCP2 interaction with GDP within the UCP2 central cavity is depicted from the ICS side (from the *top*). The structure [pdb code 2LCK; (32)] was zoomed at 20 Å sphere with basic amino acid residues responsible for binding of GDP in *red*. Color coding of transmembrane α-helices is the same as in Figure 4. GDP, guanosine diphosphate.

However, the third repeat of the GDP-bound UCP2 breaks away from this pattern (Figs. 4 and 5). The transmembrane helix H5 appears to be shifted between helices H4 and H6 toward the intermembrane/ICS side of the protein. The amphipathic helix of the same repeat rotates away by ∼45°, and its flanking regions are substantially different from their counterparts in the other two repeats. As a consequence, the matrix side of the channel is substantially more open in UCP2 than in the ANT. Thus, the ANT carrier, transporting nucleotides, has the matrix side part of a “channel” more obstructed than the UCP2 that paradoxically does not allow purine nucleotides to be translocated (Figs. 4 and 5).

### B. Structure of FA binding and anion binding site

**FIG. 6.**
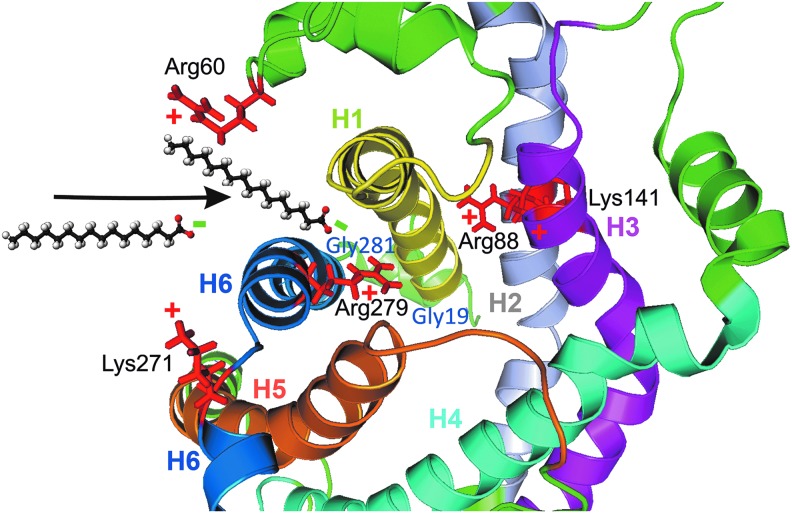
**Detailed structure of UCP2 FA binding site.** Visualization of the first phase of the FA binding to UCP2 (*arrow*) from the matrix side (from the *bottom*), zoomed at 20 Å sphere (pdb code 2LCK; (32) with depicted basic amino acids responsible for correct orientation and approaching of the FA (Arg60 and Lys271) and flipping of the FA through the cavity (Arg88, Lys141, and Arg279) in *red*. Most of the residues act also for anion transport “channel” forming with rather a large potential field (31). Gly19 and Gly281 residues (*dark blue*) are influenced by conformational changes induced by purine nucleotide binding so that they might inhibit FA^−^ anion translocation (31). Color coding of transmembrane α-helices is the same as in Figure 4.

The existence of peripheral FA binding site revealed on UCP2 strongly supports the FA cycling hypothesis (see section III.B). It was shown by NMR that FA binds to a peripheral site of UCP2 in a lipid-facing hydrophobic groove between transmembrane helices H1 and H6 and partly also to helices H2 and H5 (31) (Fig. 6). A saturable FA binding has been indicated by NMR chemical shift changes in 3D TROSY-HNCO NMR spectra in the presence of FA or long-chain alkylsulfonates. The most pronounced paramagnetic relaxation enhancement related to Leu278 is on the membrane-facing protein surface of helix 6 (31). Also 5-doxyl-C18 FA broadened a small subset of backbone resonances (31). Similarly, EPR-indicated UCP1 and 5-doxyl-C18 FA interactions have been recognized elsewhere as being affected due to the GDP-induced conformational changes (189, 195). It is confirmed (189) that GDP binding displaces the FA from its UCP1 binding site. In UCP2, the binding of GDP induces a conformational change that affects Gly281 and Gly19 residues (31). This would substantiate inhibition of the FA^−^ anion uniport.

The FA binding to the peripheral site of the UCP2 involves placing of the FA acyl chain along the hydrophobic groove between H1 and H6 and the carboxylate head group reacting with the basic residues in the vicinity. Namely Lys16, Arg60, Lys271, Lys241, and Arg279 residues participate in FA^−^ uniport mediated by UCP2 (Fig. 6) as inferred from mutations and NMR experiments (31). Note that the triple UCP1 mutant of spatially close residues C24A and D27V and T30A was fully nonfunctional, being unable to catalyze FA-induced H^+^ uniport (405).

Peripheral Arg60 and Lys271 mediate electrostatic interactions with the negative charged sulfonate group (31). Other residues form the positive potential that draws the sulfonate group into the UCP2 cavity. Among other residues, Arg 88 of UCP2 helix H2 was shown to also allow binding of alkylsulfonates besides the GDP binding (31) and was shown to be involved in chloride uniport (170). Previously, just the UCP2 helix H2 protein exerted Cl^−^ conductance patterns with clear transition between the open and closed states in single-channel current recordings by the pipette-dipping patch clamp (437). Thus, the residues inside and outside of the cavity contribute to the FA^−^ uniport as well as the uniport of other hydrophobic anions such as alkylsufonates (31, 182, 200), chloride (169), or even organic anions (415), similarly to UCP1 (183, 193, 196, 204, 208).

In conclusion, all up-to-date gathered structural data indicate that UCP2 acts as the FA flippase rather than a direct H^+^ conductor (31, 179, 181, 182, 444). Also, short-chain FAs interact with UCP2 (176). The UCP2 second translocation mode allows uniport of certain monovalent anions such as alkylsulfonates (182, 200) or pyruvate (91), and the third mode catalyzes the phosphate/aspartate antiport (or phosphate antiport with oxaloacetate, malate, malonate, and sulfate) (415).

### C. Structure of nucleotide binding site

The ATP binding site in UCP1 was found close to the proline kinks within midway of the open cavity (453). The nitroxide-labeled GDP was found closer to transmembrane helices 1–4 of UCP2 (32, 319). Consequently, GDP binding was modeled deep within the UCP2 channel (Fig. 5). Detailed inspection of the UCP2 structure bound to its inhibitor GDP showed that GDP binds inside the UCP2 cavity and that this binding can displace the FA from its peripheral site (32, 319). The antagonistic effect of GDP seems to be due to an allosteric mechanism by which GDP induces changes in conformation and/or dynamics of the H1 and H6 transmembrane helices causing disruption of the FA peripheral site (31, 182, 200).

The difference in the overall structure of UCP2 bound either to FA or to GDP is consistent with the previous observation that FA and GDP impose opposite effects on UCP2 activity (32, 319). According to the GDP binding model (32, 319), Arg 185 is in position for charge–charge interaction with GDP inside the cavity. Also cavity-lining basic residues Arg88 of the helix H2 and Lys141 of the helix H3 participate in the GDP binding (Fig. 5).

## V. Regulation of *UCP* Gene Expression

### A. *Ucp2* and *ucp3* genes

**FIG. 7.**
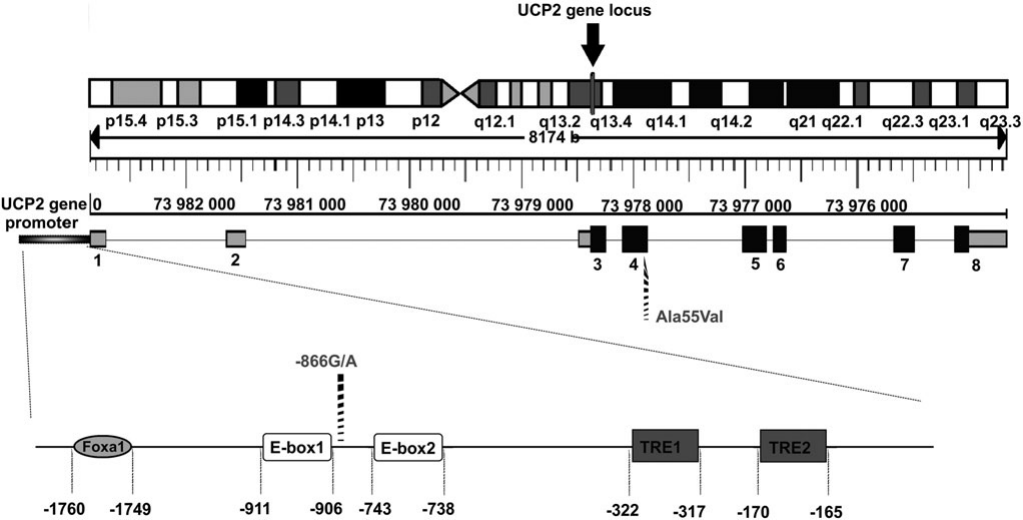
**Localization and structure of the *ucp2* gene and *ucp2* promoter with important regulatory sites**. The *ucp2* gene locus is localized in the 11q13 region of the chromosome 11 (*black arrow*). The eight exons (*boxes*) are numbered from *left* to *right* according to the transcriptional region, including the promoter region at the beginning of the sequence. The *black boxes* represent the coding regions, the *gray boxes* represent the noncoding region of the *ucp2* gene. The figure was adapted from the Ensembl genome browser for the *ucp2* gene (code ENSG00000175567). The promoter region precedes the first noncoding region of the *ucp2* gene and contains important binding sites for transcription factors such as Foxa1, E-box1 and 2 (helix-loop-helix protein binding sites), and TRE1 and 2 (thyroid hormone response elements). *Ucp2* important polymorphisms namely −866G/A and Ala55Val are depicted as well. FOXA1, forkhead box A1.

The *UCP2* gene is located on chromosome 7 of the mouse and chromosome 11 (11q13) of humans, near a region linked to diabetes and obesity (125, 142). The human *UCP2* gene spans over 8.4 kb distributed on eight exons, among them the transcription unit is made of two untranslated exons followed by six exons encoding UCP2 with the initiation site of translation in exon 3 (Fig. 7) (250, 321, 401). The mouse *UCP3* gene was mapped near *UCP2* on chromosome 7, suggesting that *Ucp2* and *ucp3* are clustered genes. This region is a boundary of synteny between human chromosomes 11q13 and 11p15. Both human *UCP2* and *ucp3* genes are assigned to chromosome 11q13 (440).

Polymorphisms within the human *UCP2* gene helped to understand its biological role in numerous diseases. Mutational analysis of the human *UCP2* gene revealed several common polymorphisms in *UCP2*: a promoter variant, −866G>A (rs659366), a missense polymorphism in codon 55 changing an alanine to a valine (Codon 55 Ala/Val, rs660339), and a 45 bp insertion–deletion polymorphism in the 3′ untranslated region (UTR) of the *UCP2* gene (3′UTR ins/del) (87, 107). The *ucp2* Ala55Val polymorphism is associated with a lower degree of uncoupling, lower energy expenditure, and, in turn, with a higher risk of obesity or higher incidence of diabetes (435). Moreover, the promoter variant (−866G>A) has been associated with obesity and/or type 2 diabetes in several studies, with the A allele having greater UCP2 expression and lower glucose-stimulated insulin secretion (GSIS) than the G allele (87, 88, 363).

The promoter variant −866G>A putatively changes one or more binding sites for transcription factors. Thus, for example, variation in the *UCP2*–*ucp3* gene cluster predicts the development of type 2 diabetes in healthy middle-aged men (129). The *ucp2* promoter polymorphism modulates lipid levels in patients with type 2 diabetes (345, 422) and the presence of the A-allele contributes to the increased UCP2, decreased ADP/ATP ratio, and decreased GSIS in glucose-tolerant subjects (368). It is associated with insulin resistance and increased risk of type 2 diabetes (85) or with increased carbohydrate and decreased lipid oxidation in juvenile obesity (247), or with a decreased risk of obesity in middle-aged humans (87, 88, 120). Cardiovascular risk in healthy men and markers of oxidative stress in diabetic men are associated with common variation in the *ucp2* gene (100). The common −866 G/A variant is associated with decreased risk of coronary artery disease in type 2 diabetic men (68). Another link was revealed for association with hypertension (212).

### B. UCP2 transcription

The mRNA of UCP2 is present in many tissues and cell types, including lung, kidney, pancreas, adipose tissue, muscle, heart, and brain (8, 125, 142) macrophages or colonocytes (175). Using two genetic mouse models of obesity, about a fivefold increase in steady-state UCP2 transcript levels relative to lean littermate controls was determined (142). Similarly, an increased expression of UCP2 mRNA was found in obesity-prone models given a lipid-rich diet relative to obesity-resistant mice (125). These initial findings, together with the hypothesized function of UCP2 in energy homeostasis and the chromosomal location of the gene, led to the hypothesis of UCP2-induced thermogenesis and weight regulation. However, this working hypothesis was gradually rejected.

Regulation of UCP2 transcription is given by the presence of several transcription factor binding sites for the specific protein-1 (Sp1), the sterol regulatory elements (SREs), the thyroid hormone response elements (TREs), and the E-box (helix–loop–helix protein binding sites) (107) (Table 1). The effectors are long-chain FAs, thyroid hormones, NAD^+^ (*via* sirtuin 1, the first of seven members of the family of nicotinamide adenine dinucleotide [NAD^+^]-dependent deacetylases), and negative regulation by TGFβ.

**Table 1.** Transcription, Translation, and Post-Translation Regulation of UCP2 and UCP3

*Transcription*				
*Gene*	*Transcription factors*	*Transcription factor binding site*	*Effectors involved in signaling cascades*	*Example of affected process*
*ucp2*	Thyroid hormone receptors	TREs	Thyroid hormone, PGC-1α	Regulation of insulin secretion
*ucp3*				
*ucp2*	Sterol response elements binding proteins	E-box 1/2	FA *via* PPARγ, PGC-1α, SREBP	Metabolism of carbohydrates and lipids
*ucp3*				
*ucp2*	Sterol response elements binding proteins	E-box 1/2	FA *via* PPARα, PGC-1α, SREBP	Acetaminophen-induced liver toxicity
*ucp3*				
*ucp2*	Sterol response elements binding proteins	E-box 1/2	PGC-1β, SREBP	Regulation of insulin secretion
*ucp2*	Foxa1	Foxa1 binding site	NAD^+^*via* sirtuin 1	Regulation of insulin secretion
*ucp2*	Sp1/3	*cis*-element	PPAR	Activation of expression in brown adipocytes
*ucp3*				

*Transcription downregulation*				
*ucp2*	SMAD binding elements	SMAD4	TGFβ	Tumor cell differentiation

*Translation*				
*mRNA*	*Translational regulators*		*Example of affected process*	
UCP2	uORF in the 5′UTR coding for a 36-AA long inhibitory peptide		Possible inhibition of UCP2 translation	
UCP2	miR-133a		Regulation by MyoD prevents UCP2 to interfere with skeletal muscle development	
UCP2	miR-15a		Repression of UCP2 translation in pancreatic β-cells and probable increase of GSIS	
UCP2	hnRNP K		Regulation of UCP2 translation as a response to insulin or adiponectin	

*Post-translational modification*				
*Protein*	*Post-translational modification*		*Example of affected process*	
UCP2	Glutathionylation		Enhancement of GSIS	
UCP3	Glutathionylation			
UCP1	Sulfenylation		UCP1 and thermogenesis activation by norepinephrine	
UCP1, UCP2, UCP3	4-hydroxyl-2-nonenal adduction		UCP activation	

FA, fatty acid; GSIS, glucose-stimulated insulin secretion; FOXOA1, forkhead box A1; NAD^+^, nicotinamide adenine dinucleotide; PGC-1α, PPARγ coactivator1-α; PPAR, peroxisome proliferator-activated receptor; Sp1, specific protein-1; SREBP, sterol regulatory element binding protein; TREs, thyroid hormone response elements; UCP, uncoupling protein; UTR, untranslated region.

Concerning FA-induced upregulation of UCP2 transcription, elevated plasma FAs increase UCP2 mRNA in white adipose tissue and various culture cells (394). FAs are ligands of peroxisome proliferator-activated receptors (PPARs), nuclear receptors that function as ligand-dependent transcription factors (114). FAs act as ligands for PPARα and PPARγ subtypes (413). The double E-box motif is required for PPARγ-dependent upregulation of UCP2 (281) such as by OleA in insulinoma INS1 cells (280). PPARγ transactivation of *ucp2* as well as *ucp3* is given by the enhancer in the *UCP3* intron 1 (50). This site transactivates the endogenous *ucp3* promoter and loops out to specifically interact with intron 1 of *ucp2*, where a weaker interaction occurs.

PPARα predetermines induction of UCP2 during acetaminophen-induced liver toxicity leading to higher ROS formation, which is counteracted by the switched on UCP2-mediated antioxidant protection (318). Dietary short-chain FAs induce a PPARγ-dependent switch from lipid synthesis to utilization in mice by decreasing PPARγ expression and activity, at elevated UCP expression and AMP/ATP ratio, consequently leading to AMPK-stimulation of oxidative metabolism in liver and adipose tissue (97). Differential modulation of AMPK/PPARα/UCP2 axis has been found in relation to hypertension and aging in the brain, kidneys, and heart of two spontaneously hypertensive rat strains (351).

The PPARγ coactivator1-α (PGC-1α) stimulates UCP2 expression mediated by thyroid hormone receptors by two TREs located in the proximal promoter (307). This regulation may be manifested during the thyroid hormone-induced reduction of insulin secretion after endurance exercise by AMPK-mediated UCP2 activation due to the increased PGC-1α (53). Also, the SRE binding protein (SREBP) upregulates UCP2, *via* either of E-box motifs upon coactivation by PGC-1β (253, 306).

A forkhead box A1 (FOXA1) transcription factor is involved in downregulation of UCP2 transcription, probably dependent on sirtuin 1 (408). Similarly, SMAD4 acts in UCP2 downregulation upon TGFβ stimulation (364). In contrast FOXA2 may be involved in UCP2 transcriptional upregulation (59).

### C. Regulation of UCP2 translation

Translational upregulation of UCP2 allows fast elevation of its protein levels under stress conditions. The first recognition of the regulation of UCP2 at translation level came from studies of starvation and LPS treatments, which increased UCP2 level up to 12 times in lung and stomach (320). However, stimulation occurred without any change in UCP2 mRNA levels, hence it should take place by translational upregulation. The UCP2 mRNA possesses a long 5′UTR, in which an upstream open reading frame codes for a 36-amino acid sequence that might hypothetically code for an inhibitory peptide, which together with 5′UTR would halt the UCP2 translation.

UCP2 expression exerted at the translational level is stimulated by glutamine at physiological concentrations (175). The upstream open reading frame in the 5′UTR of the UCP2 mRNA is required for this stimulation (174). In this way, glutamine, an amino acid oxidized by cancer cells, immune cells, pancreatic β-cells, or intestinal epithelium, is related to UCP2 beneficial functions in natural sites of its expression.

MicroRNAs turned out to be natural sequence-specific suppressors of translation blocking the expression of target protein without affecting mRNA stability. Their effect on UCP2 expression may lead to paradoxes such as the absence of detectable UCP2 protein levels at detected mRNA levels (320). For example, miR-133a regulated by MyoD prevents UCP2 to interfere with the skeletal muscle development (65). Also, miR-15a is repressing UCP2 mRNA translation in pancreatic β-cells, probably contributing to increasing GSIS (387).

The heterogeneous nuclear ribonucleoprotein K (hnRNP K) binds to UCP2 mRNA through sites located in the 3′UTR (312). Insulin might stimulate UCP2 translation *via* involvement of hnRNP K (312), similarly as adiponectin (451).

### D. Turnover of UCP2

UCP2 and UCP3 possess unusually short half-lives, which are at least one order of magnitude lower than that for UCP1. Thus, the UCP2 has a half-life of 1 h in a range of tissues (141, 350), including immortalized pancreatic β-cells where UCP2 content decreased without glutamine (19). This rapid half-life is not a general feature of other IMM carriers such as the ADP/ATP carrier and is not recapitulated in isolated energized mitochondria, suggesting that an extramitochondrial factor is required (19). This factor is represented by the cytosolic proteasome (20), as derived from studies of proteasome inhibitors, ubiquitin mutants, and a cell-free reconstituted system.

### E. Post-translational modifications of UCP2

Numerous enzymes involved in metabolism and signaling are regulated by post-translational modifications influencing their catalytic activity and rates of turnover (228). Among several modes of post-translational control exerted on enzymes (such as phosphorylation or acetylation), redox modulations may provide a feedback or strengthening of redox signaling. Cysteine residues at catalytic or allosteric sites belong to the predominant target of such redox-linked regulations (215, 228).

UCP2 and UCP3 were found to contain reactive cysteine residues that can be conjugated to glutathione (GSH). Both Cys25 and Cys259 have been identified as the major glutathionylation sites on UCP3 (267). Studies using MIN6 cells and pancreatic islets have demonstrated that induction of glutathionylation not only deactivates UCP2-mediated uncoupling but also enhances GSIS (264). Recently, the Cys253 of UCP1 has been recognized as the key regulator of nonshivering thermogenesis, being oxidized to sulfenyl by ROS upon norepinephrine stimulation of brown adipose tissue or brown adipocytes (71).

Reactive aldehydes, particularly 4-hydroxyl-2-nonenal (HNE), have been indicated to interact with UCP1, UCP2, and UCP3 (115), yet, a direct interaction from experiments studying the effect of HNE in isolated mitochondria and UCPx knockout mice has remained highly controversial (82, 370). HNE and other lipid electrophiles are highly reactive, forming covalent bonds with lysine, histidine, or cysteine residues, and protein adduction by electrophilic FA derivatives has been identified as a major outcome of oxidative stress and a redox-sensitive signaling mechanism (158, 300, 365).

The studies on the molecular mechanism of HNE action on UCPs have demonstrated that HNE does not increase proton conductance catalyzed by either UCP1 or UCP2 in the absence of FAs. Instead, the HNE binding to definite positively charged UCP amino acid residues has been suggested as a protein-mediated mechanism of the FA-dependent UCP activation, with His 217, Cys 216, and Lys 201 as candidate residues of HNE action on UCP2 (268).

### F. Regulation of UCP3 expression

The discovery of UCP3 has already identified this protein as a highly skeletal muscle-specific protein with transcript apparent also in brown adipose tissue of rodents (39). UCP3 is also minimally expressed in human heart (411). Fasting, cold, and high-fat diet typically upregulate UCP3 expression through PAR and MyoD elements (222, 383). Thus, FAs increase UCP3 mRNA in skeletal muscle and heart (139, 394), eicosapentaenoic acid and docosahexaenoic acid being quite efficient (243).

The UCP3 transcription is upregulated by PPARγ and PPARδ (413) and coactivator PGC-1α in an AMPK-mediated way (271, 348). Also an active TRE exists in the proximal promoter for *ucp3* (92, 382). In brown adipocytes, the intronic region of the *ucp3* gene contains *cis*-elements where SP1 and SP3 bind close to a direct repeat one element mediating activation of UCP3 expression by PPARγ agonists (172). The transactivation of these elements is essential for UCP3 expression, and MyoD and myogenin can also bind in their proximity similarly to recruitment of p300. The two p160 transcriptional coregulator family members SRC-1 and TIF2 modulate the UCP3 expression in an antagonistic manner (112). Also, glucocorticoids activate the UCP3 transcription through a glucocorticoid receptor-binding site in the promoter region (10).

Transcription factors may influence UCP3 transcription similarly as in *ucp2* gene, but also a different outcome can be found. For example, activation of the SREBP1 factor downregulates UCP3 in the heart of hyperinsulinemic mice (155). Activation of the JAK2/STAT3 signaling pathway also downregulates UCP3 (255). Recently, estrogen was found to downregulate UCP3 (293) and vitamin D3 to upregulate UCP3 by binding into its promoter region (123).

### G. Regulation of UCP4 and UCP5 expression

Regulation of gene expression for UCP4 has to be strict to allow UCP4 expression only in neurons and in certain other cell types such as chondrocytes (173). UCP4 is simultaneously upregulated together with typical neuronal marker proteins TUJ-1 and NeuN during mouse embryonic stem cell differentiation *in vitro* as well as during murine brain development (355). Also, overexpression of c-Rel downstream of NFκB pathway increases *ucp4* promoter activity and protein expression (168, 167, 239, 341, 430). Not much is known for UCP5 transcription, neither its tissue distribution for which mRNA is irrelevant due to possible translational downregulation. Originally UCP5 mRNA has been found to increase with a high-fat diet in the liver but not in the brain (443).

## VI. Redox Homeostasis and Mitochondrial and Cell Redox Regulations

### A. Mitochondrial redox state frequently regulates cellular redox state

#### 1. Distinct nature of mitochondrial ROS sources

**FIG. 8.**
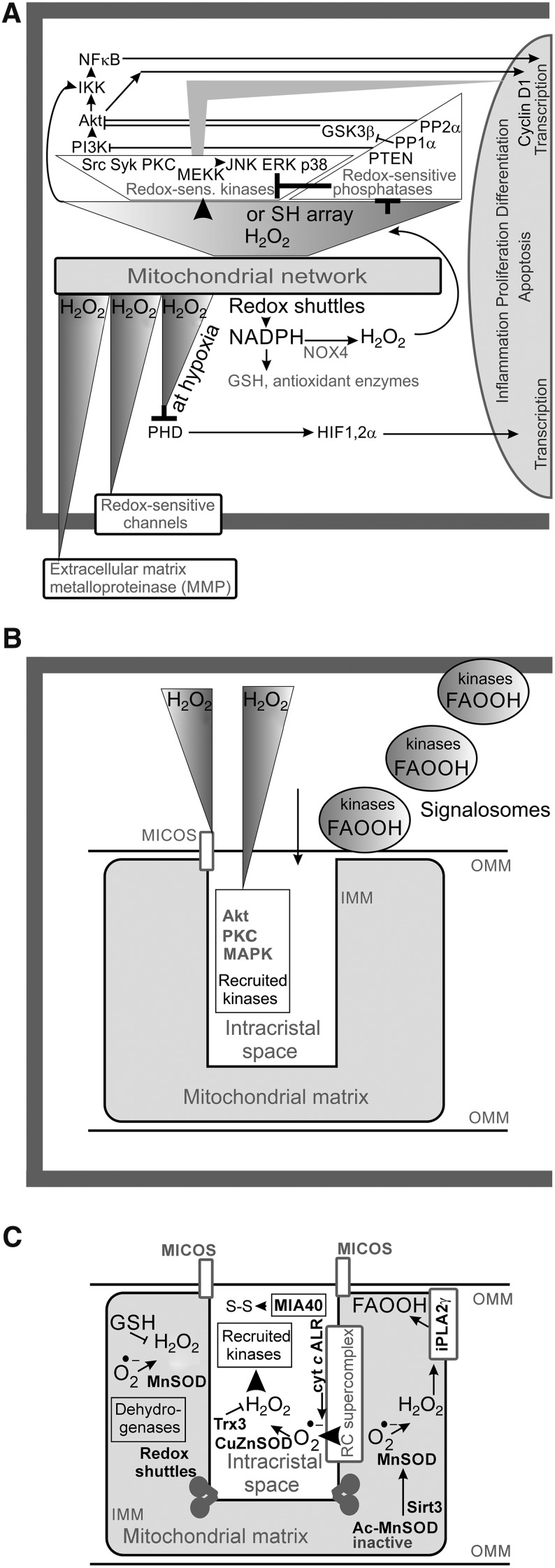
**Three types of redox signaling.** **(A)** Retrograde redox signaling from the mitochondrion directed toward the cell cytosol, nucleus, plasma membrane, or other cell components is mostly executed *via* the H_2_O_2_ diffusion or by redox relaying enzymes (329). Besides redox-sensitive kinases and phosphatases (*upper part*), the prominent receivers of the redox signal are extracellular MMP, redox-sensitive channels, and upon hypoxia also PHD enzymes, which are inhibited, similarly as the FIH, both leading to HIF1-α accumulation and resulting HIF-mediated transcriptome reprogramming. **(B)** External redox signaling from the cell toward the mitochondrion including H_2_O_2_ activation of kinases within the ICS and hypothetical plasma membrane derived signalosomes (133, 336); likewise, redox signaling originating from norepinephrine stimulation of brown adipocytes leading to sulfenylation of Cys253 in UCP1 serves as a clear example (71), and **(C)** intramitochondrial redox signaling of a short range exists just within the interior of the OMM, forming tubules of mitochondrial network reticulum. A continuous compartment is represented by the matrix space, which is interrupted by the rich cristae. The cristae lumen called ICS represents numerous separate compartments, which are interconnected only *via* the crista outlets and the outer intermebrane space (a *middle part* of the sandwich of OMM and IMM). Within the matrix, typical redox regulation is exerted by acetylation of MnSOD, making it inactive, and deacetylation by sirtuin-3, activating MnSOD (390). Also, H_2_O_2_-activated PNPLA8 (iPLA2γ) (180, 186) is able to cleave FAOOH (254, 288), which are both substrates of UCP2-catalyzed H^+^ transport (181) and signaling molecules (162, 299, 300), leading to separate redox-sensitive signaling pathways. Within the ICS, reducing system is represented namely by CuZnSOD and thioredoxin-3 (Trx3). Within the outer intermembrane space (or hypothetically also in ICS), also MIA40/ALR SH-oxidizing protein system shifts the local environment to more prooxidant state (206). All these systems are fed by superoxide from the distinct source, the site III_Qo_ of complex III, under retardation of cytochrome *c* shuttling (*e.g.*, upon hypoxic ALR regeneration) and upon hypoxia (376, 426). *Arrows*: activation; *half-open line segment*: inhibition. Akt, protein kinase B; FAOOH, FA hydroperoxides; FIH, factor inhibiting HIF; HIF1-α, hypoxia-inducible factor 1-α; GSH, glutathione; MAPK, mitogen-activated protein kinase; MMP, matrix metaloproteinases; MnSOD, superoxide dismutase 2; NOX, NADPH oxidase; OMM, outer mitochondrial membrane; PHD, proline hydroxylase domain; PKC, protein kinase C.

Mitochondria in numerous tissues represent a major source of superoxide and subsequent downstream oxidants, notably H_2_O_2_ (43, 44, 109, 338, 403). Since mitochondrial participation on the whole redox homeostasis in the majority of cells is high, UCPs can influence even extracellular function *via* transferring redox state from the insulated matrix/ICS compartment of mitochondrion. H_2_O_2_ released from or to mitochondria can significantly influence redox homeostasis in both the cytosol and mitochondrial matrix (61, 198, 329). The mitochondrial production of superoxide is not devoted only to a wasteful buildup of oxidants leading to oxidative stress and impairment of cellular housekeeping, but is frequently employed for the retrograde redox signaling, directed as information for the cell cytosol, nucleus, plasma membrane, or other cell components (Fig. 8A). Such *retrograde redox signaling* reports on either bioenergetics/metabolic state or has been incorporated by evolution to modulate particular physiological phenomena (Table 2), such as GSIS (186). Retrograde signaling is implicated in pathology (237) and exists also in plants and chloroplasts as recently reviewed in Ref. (225).

**Table 2.** Redox Signaling with UCP2 or UCP3 Participation

*Signaling type*	*Tissue, cell type*	*Protein/activity up/down*	*Redox signaling*	*Process*	*Stimulus/concomitant response*	*References*
ROS/MAPK (cJun, p38)	Macrophages	UCP2 protein down	On	LPS-induced ROS signaling	LPS	(118)
MAPK (p38, ERK1,2) suppression	Macrophages	UCP2 protein up	Off	Impaired ROS response to parasite (release of ROS-mediated inhibition of protein tyrosine phosphatases)	*Leishmania*	(26)
MAPK (ERK)	Erythrocyte progenitor cells	UCP2 protein up	Off	Facilitation of heme synthesis by ROS reduction	Erythropoietin	(117)
ROS/cJun suppression	Dermal fibroblasts	UCP2 activation	Off	Attenuation of ROS formation	Aging	(69)
GLP1/AMPK	Endothelial cells (aortic)	UCP2 protein up	Off	Inhibited COX2 expression	GLP1	(256)
Akt suppression	Keratinocytes	UCP3 protein up	Off	Block of skin carcinogenesis		(304)
PTEN-induced putative kinase-1	Endothelial cells	UCP2 protein down	On	Redox signal promotion		(156)
Akt, PKC, MEK	Immune, glial cells	UCP2 protein up	Off	Inhibition of proinflammatory cytokines and activation of cell survival factors		(151)
Leptin	Orexigenic neurons	UCP2 activation	Off	Homeostat, “leptin resistence”	Leptin	(13, 78)
Propiomelanocortin	Orexigenic neurons	UCP2 activation	Off	Glucose sensing	Glucose	(230, 317)
	Hypothalamic neurons (vntrm.nuclei)			Glucose sensing	Glucose	(395)
Suppression of NLRP3 inflammasome activation	Endothelial cells	UCP2 protein up	Off	Ghrelin-stimulated suppression of inflammation induced by LDL	Ghrelin	(353)
Suppression of NLRP3 inflammasome activation	Astrocytes	UCP2 protein up	Off	Prevention of ROS- mediated NFkB stimulation of NLRP3 and TXNIP stimulation of NLRP3 inflammasome	Prevention of depression	(110)
Yap	Embryonic neurons	UCP2 protein up	Off	Ubiquitination and degradation of Yap	Neurogenesis	(211)

Akt, protein kinase B; LDL, low-density lipoprotein; MAPK, mitogen-activated protein kinase; NLRP3, NOD-, LRR,- and pyrin domain-containing protein 3; PKC, protein kinase C; ROS, reactive oxygen species; TXNIP, thioredoxin-interacting protein.

In contrast, a huge influence of *external redox signals* exists, emanating from the cell cytosol toward mitochondrion, thus executing the redox signaling from the cell to mitochondrial components (Fig. 8B), which is often relayed by kinases or manifested as post-translational regulations. Finally, redox regulations of a short range exist just within the mitochondrion (Fig. 8C), that is, within the compartment limited to the interior of the OMM, forming tubules of mitochondrial network reticulum. A typical example of recently found redox switch is sirtuin-3-mediated deacetylation of superoxide dismutase 2 (MnSOD), which is inactive in the acetylated state (398).

**FIG. 9.**
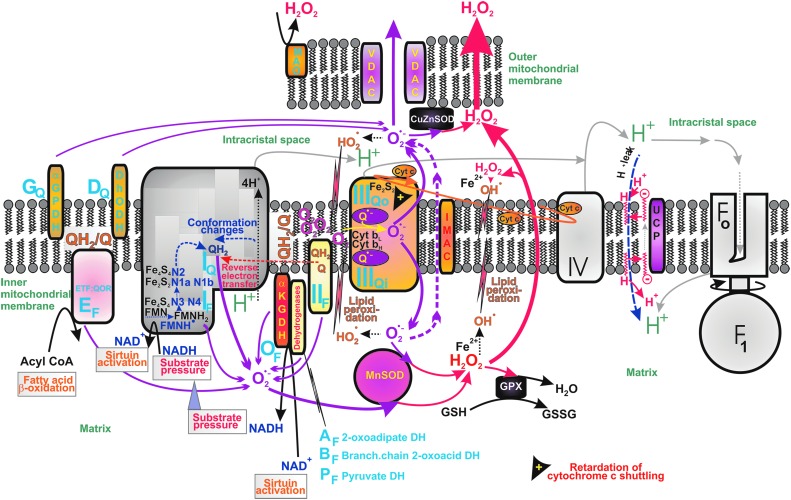
**Sites of mitochondrial superoxide production.** Overview of locations for superoxide sources (*blue capital fonts*) is illustrated, while their assumed relative contribution is expressed by the arrow thickness (*purple arrows* for superoxide, *red arrows* for H_2_O_2_). The sites of superoxide formation are termed according to the nomenclature introduced by Martin Brand (43), includes six sites acting at the ∼280 mV redox potential of the NADH/NAD^+^ isopotential pool (*index F, flavin*) and five sites acting at the ∼20 mV redox potential of the ubiquinol/ubiquinone (QH_2_/Q) isopotential pool (*index Q*). Among them, only the sources depending on Δ*p* (Δ*Ψ*) and relying on the smoothed (unretarded) process of forward electron transport within the respiratory chain can be directly attenuated by uncoupling. Specifically, these uncoupling-attenuated sources ensure superoxide formation at the site I_Q_ of complex I and site III_Qo_ of complex III. In turn, the complex I site I_F_ increases superoxide formation at NADH >> NAD^+^ (at a higher substrate pressure NADH/NAD^+^). Typically, in pathological conditions, allowing reverse electron transfer to the complex I site I_Q_, this site produces the majority of superoxide; when pathology retards cytochrome *c* shuttling (*orange elliptic arrow*), the complex III site III_Qo_ provides major superoxide formation. The latter superoxide formation is not attenuated by uncoupling. αGPDH, α glycerolphosphate dehydrogenase; αKGDH, α ketoglutarate dehydrogenase; DH, dehydrogenase; DhODH, dihydroorotate dehydrogenase; ETF:QOR, electron-transferring-flavoprotein:ubiquinone oxidoreductase; MAO, monoaminooxidase; NAD^+^, nicotinamide adenine dinucleotide.

In mitochondria, there are numerous sites known to generate superoxide (Fig. 9). The basal rate of superoxide formation proceeds inevitably within the respiratory chain under all conditions. Superoxide is dismuted by matrix MnSOD and ICS/intermembrane space CuZnSOD to H_2_O_2_, freely permeant through the mitochondrial and cell membranes (64, 391) or through yet mostly putative IMM aquaporins (see section VI.B). Hence a suddenly elevated mitochondrial H_2_O_2_ release may substantiate redox regulations toward the cell cytosol and other compartments, termed retrograde redox signaling.

Concerning superoxide formation, together with complex I and complex III of the respiratory chain, there are at least eight other sites of superoxide formation on dysfunctional succinate dehydrogenase (complex II) and other intact matrix 2-oxoacid dehydrogenases, including pyruvate dehydrogenase (305) (plus glycerol phosphate and dihydroorotate dehydrogenase oriented and releasing superoxide toward the cytosolic side, *i.e*., ICS). The reader can refer to thorough reviews on this subject (43, 44, 109, 338). Note, however, since these superoxide-generating enzymes are only indirectly coupled to proton pumping and respiratory chain electron transfer, the uncoupling by UCPs can only indirectly influence such superoxide production.

Among 11 different sites recognized to generate superoxide in isolated mitochondria, 6 sites act at the redox potential of the NADH/NAD^+^ isopotential pool (∼280 mV; denoted by the index F in Fig. 2, including the complex I site I_F_) and 5 sites acting at the ∼20 mV redox potential of the ubiquinol/ubiquinone (QH_2_/Q) isopotential pool, including the complex I site I_Q_ and the complex III site Q_o_ (denoted by index Q in Fig. 9) (43). Nevertheless, their contributions to the overall mitochondrial superoxide in the matrix or intracristal compartments and to downstream ROS are different for the different cell types and metabolic modes (262, 265, 375). Also, complex ROS homeostasis in intact cells cannot be directly derived from that observed in isolated mitochondria.

For complex I of the respiratory chain, it has been recognized that at higher substrate pressure (higher NADH/NAD^+^), the superoxide formation is elevated at the flavin in the NADH-oxidizing site (site I_F_) (187, 399). Such superoxide elevation may turn into a redox signal directed toward the matrix space, because the site I_F_ is oriented toward the matrix. Alternatively, when metabolic conditions allow for the reverse electron flow, the elevated complex I superoxide formation now proceeds at the ubiquinone-binding site (site I_Q_), similarly as this proceeds in the rotenone-inhibited complex I (187, 399). This superoxide increase might substantiate a redox signal also directed toward the matrix.

Another significant producer of superoxide is complex III at the site of quinol oxidation (“outer” site III_Qo_, embedded within the outer IMM phospholipid leaflet facing the ICS). For example, under conditions when cytochrome *c* shuttling is retarded, higher superoxide production rate at complex III may create the redox signal (109). Since superoxide produced by complex III is released about equally to both the matrix side and to the cytosolic side (predominantly to the ICS), the concomitant redox signaling can be directed also to the cell cytosol. This signaling has been well characterized under the conditions of hypoxia. Hypoxic signaling is the best documented retrograde signaling from mitochondria to the cell cytosol, where it deactivates the proline hydroxalase domain enzymes and thus initiates, for example, hypoxia-inducible factor 1-α (HIF1-α) stabilization with concomitant HIF-mediated transcriptome reprogramming (23, 30, 62, 207, 272).

#### 2. Uncoupling as a mechanism downregulating mitochondrial superoxide formation

**FIG. 10.**
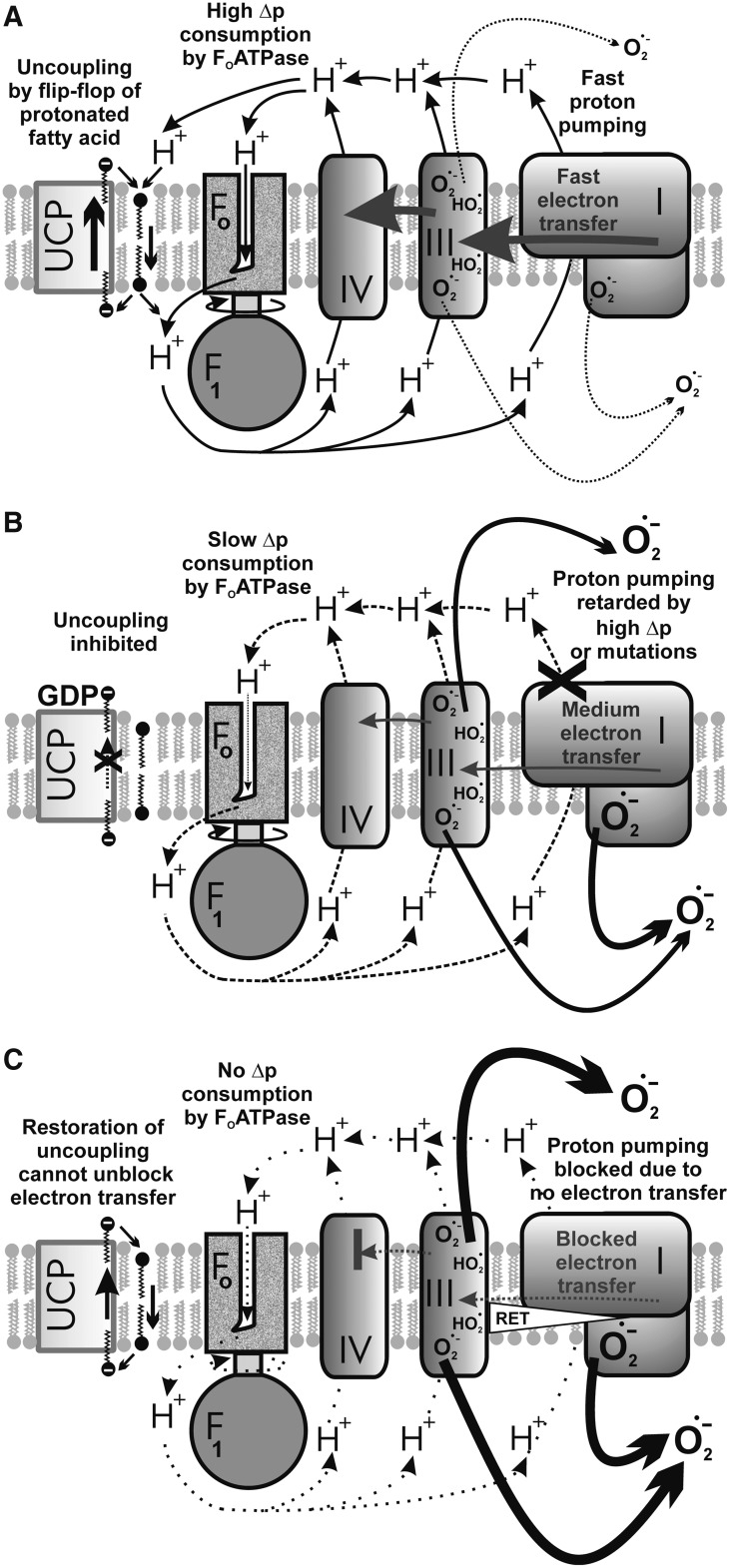
**Influence of mild uncoupling on superoxide production.** **(A)** Higher rates of respiration and proton pumping promoted by mild uncoupling attenuate superoxide formation—specifically at complexes I and III. **(B)** Regulated UCP inhibition/inactivation at simultaneous maximum ATP synthesis reaches so-called respiratory control establishing the slow electron transfer (respiration) and slow proton pumping, both promoting higher superoxide formation namely at complexes I and III. **(C)** Electron transfer blockage or slowdown such as resulting from retardation of cytochrome *c* cycling between complexes III and IV cannot be influenced by uncoupling and typically leads to a high complex III superoxide formation at site III_Qo_. Alternatively, at pathological succinate accumulation leading to reverse electron transfer (“RET”), a high superoxide formation exists at the I_Q_ site of complex I. *Thicker arrows* indicate higher fluxes, *dashed lines* represent medium fluxes, and *dotted lines* represent the absence of fluxes. *Orientation*: *bottom parts* represent the matrix; *upper parts* represent the ICS.

Theoretically, the influence of mild uncoupling onto Δ*Ψ*_m_ can be considered as (i) direct and (ii) indirect. The direct effects represent the inevitable direct Δ*p* or Δ*Ψ*_m_ influences onto the superoxide sources, whereas the indirect effects stand for mediation by lower substrate pressure due to retardation of metabolite transport processes dependent on ΔpH or Δ*Ψ*_m_, including the redox shuttles. The main feature of mild uncoupling lies in the fact that it promotes slightly higher rates of respiration, that is, higher rates of proton pumping and electron transfer, which, in turn, do not allow high superoxide formation rates (Fig. 10A). In contrast, at maximum ATP synthesis reached at maximum state-3 and at simultaneous absence of UCP-mediated uncoupling, the resulting respiratory control establishes the slow electron transfer (respiration) and slow proton pumping. Both promote higher superoxide formation (Fig. 10B).

It has long been postulated that a mild uncoupling, a partial dissipation of Δ*p*, is involved in the cellular defense system preventing mitochondrial superoxide formation (374). In line with the postulate, it has been observed in isolated mitochondria that the rate of mitochondrial superoxide/H_2_O_2_ production (given mostly by the reverse electron transfer in these pioneer experiments) decreases exponentially with decreasing Δ*Ψ*_m_ (232, 233) and is closely related to the NAD(P)H redox state (384). The ability of mild uncoupling to decrease Δ*Ψ*_m_-dependent superoxide formation is pronounced under the conditions of reversed electron flow/transfer, which results at superoxide formation at the I_Q_ site of complex I (73, 103).

As a note, the dissipation of Δ*p* cannot attenuate superoxide formed as a result of cytochrome *c* cycle retardation on complex III (Fig. 10C). In addition, when the complex I proton pumping is inhibited, for example, by mutations of mtDNA-encoded ND subunits, uncoupling is ineffective in decreasing the resulting elevated superoxide formation (105). The question remains whether such uncoupling might have some influence on the superoxide formation of broken preapoptotic structure of crista outlets. With the majority of cytochrome *c* unreleased, one may predict a certain influence, such as judged from Δ*Ψ*_m_ dependencies of isolated mitochondria, however, with the majority of cytochrome *c* released, its predominant absence maintains high superoxide formation that cannot be diminished by uncoupling. Nevertheless, given the numerous experimental pieces of evidence reviewed hereunder, we conclude that during selected physiological situations, mild uncoupling attenuates mitochondrial superoxide formation (Fig. 10A, B).

**FIG. 11.**
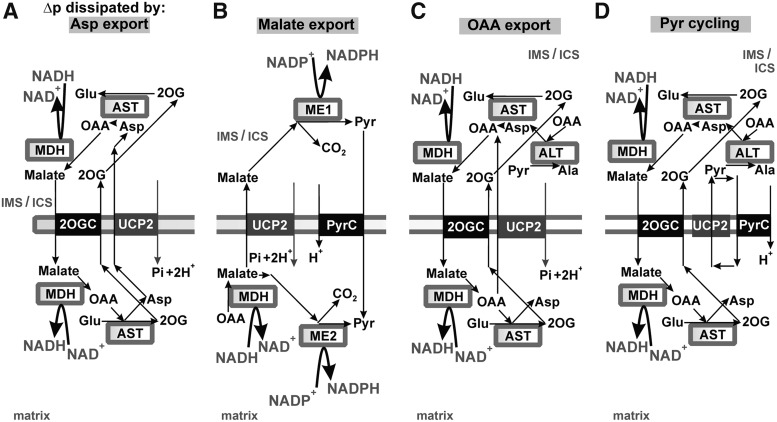
**UCP2-mediated anion efflux from the matrix may substitute metabolite carriers within redox shuttles.** Such efflux is driven by the Δ*Ψ*_m_ component of Δ*p* (similarly to the ATP^3−^/ADP^2−^ antiport enabled by the ADP/ATP carrier), hence partially dissipates Δ*p*. **(A)** Aspartate export (“Asp”) in a synergy with the 2-oxoglutarate carrier (“2OGC”) substituting the malate aspartate carrier within the malate/aspartate redox shuttle. **(B)** Malate export (“Asp”) in a synergy with the pyruvate carrier (“PyrC”) may enable so-called pyruvate/malate redox shuttle with participating cytocolic malic enzyme 1 (“ME1”) and matrix ME2. **(C)** Oxaloacetate export in a synergy with the 2-oxoglutarate carrier. **(D)** Pyruvate cycling dissipating whole Δ*p*, enabled by the UCP2-mediated pyruvate uniport and pyruvate proton symport by the pyruvate carrier. Δ*Ψ*_m_, mitochondrial inner membrane potential; ALT, alanine aminotransferase; AST, aspartate aminotransferase; MDH, malate dehydrogenase; OAA, oxaloacetate.

Recently, a second mode for UCP2 influence onto redox homeostasis independent of its uncoupling activity has been suggested (415): by enabling aspartate, malate, or oxaloacetate export from the matrix in exchange for the incoming phosphate (see section VII.A). At first, Δ*Ψ*_m_ dependence is expected for the antiport of divalent C4 anion with the monovalent biprotonated phosphate, likely ongoing in intact cells in contrast to the electroneutral antiport found in proteoliposomes (415). Likewise, the interference with redox shuttles transferring redox equivalents of NADH (NADPH) may be established by such antiport mode of UCP2 (Fig. 11). We discuss these possibilities hereunder.

Since as already noted, mitochondrial superoxide formation is elevated at higher Δ*p* (35, 103, 147, 273, 337), its attenuation by uncoupling is theoretically feasible. Mostly, Δ*Ψ*_m_ dependence of mitochondrial superoxide formation is considered also for intact cells. Thus HepG2 cell superoxide formation is enhanced at higher retardation of proton pumping by complex I (104, 105). The native ultimate force retarding physicochemically proton pumping is Δ*p*, standing behind the defined respiratory control in mitochondria. Thus also for complex I at higher Δ*p*, meaning also at higher Δ*Ψ*_m_, superoxide formation is higher. Consequently, complete uncoupling (setting Δ*p* to zero) diminishes also superoxide formation therein.

Simulating proton pumping retardation within complex I (such as caused by mutations of, for example, ND5 mitochondrion-encoded subunit) by blockage with an amiloride derivative, we have found elevated superoxide formation; however, this blockage could not be overcome by uncoupling (105). As a consequence, mitochondrial uncoupling cannot attenuate superoxide formation caused by mutations of mitochondrion-encoded ND subunits. Indeed, this has been experimentally observed by Kukat *et al.*, who showed that upregulating UCP2 in mtDNA mutator mice is not associated with altered proton leak kinetics or ROS production (238).

#### 3. Attenuation of superoxide formation by UCPs

A mild uncoupling is physiologically provided by UCP2 to UCP5. The fact that phylogenesis generated these proteins turned out into an advantage of having a repertoire of the regulated uncoupling. Owing to the relative predominance of mitochondrial ROS source within the cell, one can predict that even accumulated oxidative stress might be attenuated by long-term mild uncoupling. The theoretically well-developed concept of uncoupling-dependent attenuation of mitochondrial superoxide formation (45, 198, 233, 266) has been, however, experimentally verified with certain difficulties or doubts during the years (54, 352, 369). Here we summarize the seminal supporting findings.

Regulations of redox homeostasis by UCP-mediated uncoupling have been implicated in numerous physiological and pathological situations, including aging (12). They are subjects of a detailed review in section VIII.

Soon after the discovery of UCP2, Nègre-Salvayre *et al.* (294) reported a suppression of hydrogen peroxide production due to the UCP2 function. A support for ROS attenuation due to uncoupling has been reported for UCP2(^−/−^) mice, which were more resistant to *Toxoplasma gondii* infection due to the higher macrophage attack (17). Indeed, the lack of oxidant-attenuating capacity within mitochondria amended literally a higher strength for extracellular oxidants, which were superimposed to the classic macrophage-activated NADPH oxidase (NOX) superoxide formation. Today, we may amend the interpretation in terms of anti-inflammatory function consistent with the findings by Emre *et al.* (118). Similarly, UCP3(^−/−^) mice exhibited higher levels of ROS in skeletal muscle (412). UCP3 antioxidant role has been demonstrated in isolated skeletal muscle mitochondria (396). Duval *et al.* (113) have demonstrated a role for UCP2 in control of superoxide production and subsequent oxidation of the surrounding compounds mediating oxidative stress of endothelial cells. Mice with deleted low-density lipoprotein receptor exhibited extensive diet-induced atherosclerotic plaques when they received bone marrow transplanted from UCP2(^−/−^) mice, and the appearance of these plaques was prevented when they received bone marrow transplants from UCP2 (+/+) mice (34).

Specifically, UCP2 exerts an important antioxidant role in pancreatic β-cells (246, 327), preventing excessive superoxide formation within the respiratory chain (347). The UCP2 antioxidant cytoprotection has been documented in detail for insulinoma INS1E cells (24, 186). Recently, we have demonstrated that UCP2 provides a significant attenuation of matrix-released superoxide in model β-cells solely in the presence of iPLA_2_γ, indicating that nascent FAs cleaved by iPLA_2_γ are essential for the UCP2-mediated protonophoretic function (186). Moreover, since mitochondrial FA β-oxidation produces superoxide and subsequent hydrogen peroxide, this can activate the redox-sensitive iPLA_2_γ and hence also initiate uncoupling *via* UCP2 (186) and hypothetically other UCPs including UCP1 in brown adipose tissue.

A wide impact of UCP-mediated antioxidant protection in cell physiology can be illustrated in other examples. UCP2 has been indicated to mediate a significant antioxidant protection in human mesenchymal stem cells (36). Shorter lives of UCP2-deficient mice also stem from a profound antioxidant protection besides deteriorated insulin/IGF-1 signaling (164). The defense mechanism by counteracting the excessive ROS production was found in L6 myocytes stressed by docosahexaenoic acid after upregulation of UCP2 or UCP3 expression (57).

A self-standing topic is represented by the attenuation of superoxide production by UCP2 in cancer cells. UCP2 overexpression in colon cancer cells resulted in diminished apoptosis (caspase-3 activation) also *via* suppressing phosphorylation of p53 (99). UCP2 inhibition produced oxidative stress and sensitized breast cancer cells to therapeutic agents (332). Thus, a preventive role of UCPs in oncogenesis is considered. Finally, neuronal-specific UCP4 and UCP5 also play an important role in neuron antioxidant defense and neuroprotection (167, 168, 239, 341, 430). UCP2 antioxidant role has even been indicated for rat retina in the early stages of diabetes (311). Other examples of UCP2 antioxidant role are discussed in section VIII.

### B. Mitochondrion as major hub for cell redox signaling

The mitochondrion (the *single network of mitochondrial reticulum* in intact cells) or mitochondria (*fragments* of the network when fission prevails over fusion) are significant sources of superoxide and other ROS (198, 329). Importantly, superoxide formation depends on metabolic states and umbrella of physiological regulations, which are sophistically integrated into the redox states and in numerous cases serve for the generation of information redox signals. This is typically provided by a transient increase in steady-state H_2_O_2_ concentrations after superoxide conversion by matrix MnSOD or intracristal CuZnSOD. Also superoxide oscillations have been reported, based on the opening of the poorly characterized inner membrane anion channel (14, 15, 38, 190). Besides the direct ROS transfer from the mitochondrion to the cytosol or other cell compartments, also transfer of redox equivalent in the form of NADH and NADPH participates in parallel balance of redox homeostasis or counteracts it. The respective redox shuttles are beyond the scope of our review [see, *e.g.*, Ref. (214)]. One must also point out the role of nicotine amide nucleotide transhydrogenase, normally phosphorylating matrix NADH to NADPH on the expense of Δ*p*, which can be, however, reversed under pathological conditions (298).

Diffusion of superoxide and H_2_O_2_ or other ROS within the topologically complex matrix continuous compartment and separate ICS compartments (329) strongly depends on the presence of redox buffers therein. In the matrix and ICS, MnSOD and CuZnSOD activities, respectively, are balanced by activities of H_2_O_2_ removing enzymes (ICS being more shifted to a prooxidant state). Thus the matrix GSH redox cycle in parallel with MnSOD-mediated scavenging of superoxide is crucial for preventing excessive H_2_O_2_ accumulation. Consequently, cells with low capacities of redox buffers such as pancreatic β-cells spread redox signals much more readily.

Situation is more complex in cells at a dormant OXPHOS state when MnSOD is inhibited by acetylation. In their conversion into the pro-OXPHOS state, sirtuin-3 (−4,-5)-mediated deacetylation reactivates MnSOD (390). Such reactivation on one hand attenuates peaking superoxide formation (if it occurs), but on the other hand, it initiates new H_2_O_2_ emanation that may serve as a redox signal. Also, detailed discussion of other redox players is out of scope of this review, such as concerning GSH transferases (specific mitochondrial isoforms [GSTα1-1, GSTα4-4, and GSTμ1-1] and a specific mitochondrial thioredoxin isoform [Trx2]), acting in concert with peroxiredoxins Prx5 and Prx3 (198).

Also, mitochondrial ROS formation can be amplified by cytoplasmic NOXs and *vice versa*. ROS-mediated activation of NOX by mitochondrial ROS in liver macrophages and endothelial cells is manifested during systemic inflammatory response, leading to liver failure (237). Activation of NOX in pulmonary artery smooth muscle cells under hypoxic conditions also requires a mitochondrial ROS source (331, 342). The other direction in a crosstalk between NOXs and mitochondrion (86) can be exemplified by the following revealed regulatory pathway in endothelial cells: NOX4-derived H_2_O_2_ activates NOX2, which *via* pSer36-p66Shc signaling increases mitochondrial ROS production and furthemore enhances VEGFR2 signaling and angiogenesis (223).

During redox signaling, an elevated superoxide formation is directed either to the mitochondrial matrix or intracristal space lumen. The former is, representing the continuous matrix tubule and hence provides a delocalized integral H_2_O_2_ source for the cell cytosol. Indeed matrix MnSOD promptly catalyzes the reduction of the majority of superoxide into H_2_O_2_.

A more localized superoxide release exists to the intracristal lumen and is manifested to a higher extent when crista outlets are widened or broken (329). The typical example is hypoxic redox signaling, leading to the activation of hypoxia-induced factor (HIF) (30, 62, 152, 328, 376, 426). Most frequently, redox signaling is relayed to numerous signaling processes. Thus, besides the HIF-1α activation, the hypoxic signaling is initiating the carotid body hypoxic response, activates AMPK, NFκB, ensures the endothelial cell phenotypic response, and participates in hypoxic pulmonary vasoconstriction (376, 426). Speculatively, UCP (over)activation might attenuate these responses, whereas UCP downregulation or inhibition might amplify them.

Final acceptors of transiently elevated ROS levels are, for example, kinases, which are activated, and phosphatases, which are usually inhibited by H_2_O_2_ (Fig. 1A). Proteinases and metaloproteinases are also redox activated (447). H_2_O_2_ may spread by diffusion within the proximal cell compartments, or, alternatively, the redox relay mechanistically occurs by oxidizing critical cysteine residues not only in the final acceptor proteins but also during the signal transmission by redox transfer proteins (372). The integrated network of thiols constitutes a regulatory device involved in the maintenance of steady-state H_2_O_2_ levels, mitochondrial and cellular redox, and metabolic homeostasis (439). Consequently, redox regulations through redox switches of protein cysteine thiols involve reversible oxidation by H_2_O_2_ or superoxide of the cysteine thiol to sulfenic acid (58, 244) or a sulfenic acid loop.

The latter exists, for example, in the catalytic site of glutaredoxins (Grx1 and Grx2). Cysteine residues are part of the iron–sulfur (Fe-S) assembly, heme and zinc finger motifs, which are also abundant in mitochondria and thus the mitochondrial proteome is very rich in protein thiols. Moreover, redox potential is influenced by pH. Thus, alkaline pH within the mitochondrial matrix lowers the redox potential even more, increasing the protein reactivity. In turn, a local low ICS pH does the opposite.

Emanation of redox signaling by H_2_O_2_ is also facilitated by aquaporins AQP3, AQP8, and AQP9, mostly in plasma membrane, which is important for extracellular redox signaling (279). Downregulation of aquaporin expression then decreases extracellular H_2_O_2_ signaling found in immune, kidney, and neurons (33, 248, 425). In keratinocytes, AQP3 is required for NF-κB activation ongoing, for example, in the development of psoriasis (154). In mitochondria, AQP8 contributes to cell viability (270). However, it should be further determined what portion of H_2_O_2_ fluxes across mitochondrial membranes is mediated by aquaporins.

### C. Hypothetical assumptions for UCP participation in redox signaling

Given the ability of mitochondrial uncoupling to attenuate the mitochondrial superoxide production, we can postulate that switch-on/switch-off regulations of UCPs will profoundly affect cell redox homeostasis, specifically at the low capacity of cell redox buffers, for example, at low GSH. At first, UCP function can modulate the ongoing redox signaling in the cell cytosol originating from nonmitochondrial sources.

*Switching on UCP* function should *attenuate* cytosolic or extracellular matrix (ECM) redox signals, since the resulting diminished mitochondrial superoxide formation upon mild uncoupling releases the additional antioxidant capacity, either within mitochondrion or cell (198). Consequently, the ongoing redox signal possesses very low amplitude. This can be regarded as *redox signal termination or attenuation*. Owing to the same principle, the *switching off* the UCP-mediated mild uncoupling will *increase the amplitude* of the cytosolic or ECM *redox signal*, since the enhanced mitochondrial superoxide formation is amended to the total redox burden. Moreover, the capacity of cytosolic or ECM redox buffers is now partly occupied by such enhanced mitochondrial superoxide formation, specifically by the portion that radiates to them (mostly in the form of H_2_O_2_), hence redox signaling may spread easily.

**FIG. 12.**
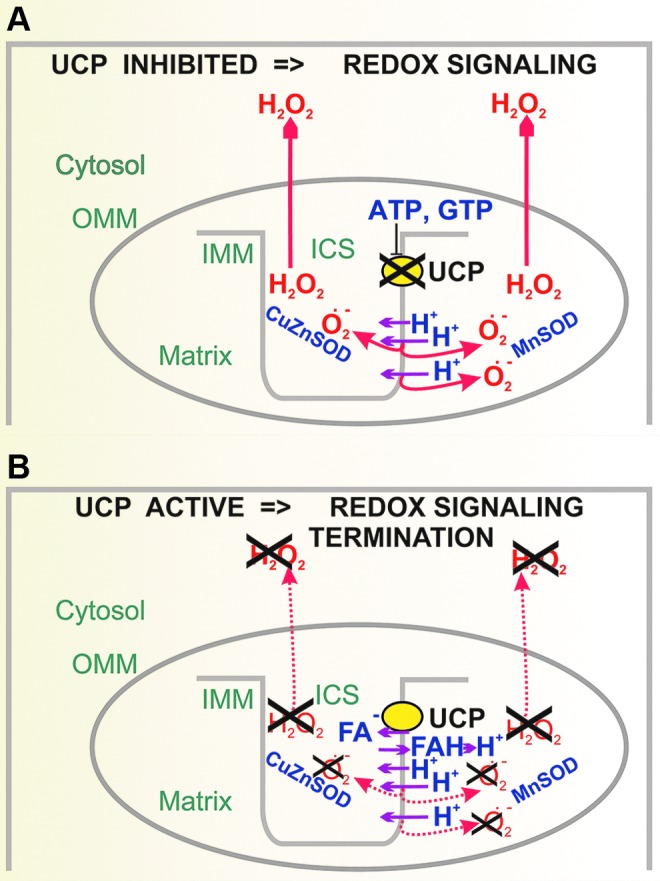
**Roles for UCPs in cellular redox signaling**. **(A)** Switching off the UCP protonophoretic function initiates novel mitochondrial retrograde or internal redox signaling by directly increasing mitochondrial superoxide production, or else promotes the ongoing cell redox signaling by decreasing the capacity of cell antioxidant systems that had to buffer the excessive mitochondrial ROS arising due to the absence of UCP2-mediated antioxidant protection. Primary superoxide is reduced to the signaling H_2_O_2_ by means of MnSOD (in the matrix) or CuZnSOD (in the ICS/intermembrane space). **(B)** Switching on the UCP protonophoretic function terminates the redox signaling by directly decreasing of mitochondrial superoxide production or attenuates cell redox signaling by increasing the spare capacity of cell antioxidant systems. ROS, reactive oxygen species.

The described principles are valid even when the redox signaling is arising from the mitochondrion. However, the UCP itself can be a primary signaling initiator and as such UCP participates in the redox signaling directly. Again, there are two views explaining the initiation as well as the termination of the redox signal (*i.e.*, consensually prooxidant event) stemming either from the direct influence of UCPs on mitochondrial superoxide formation or from shifts of the whole cell redox homeostasis (Fig. 12) (198, 329). One can predict that

(i) redox signaling is initiated by switching off the UCP protonophoretic function, which releases the extra contribution of mitochondrial superoxide sources previously attenuated by mild uncoupling. Such surplus mitochondrial superoxide released to the cell cytosol substantiates the newly created redox signal, specifically after superoxide dismutation into H_2_O_2_.
(ii) An alternative view points out that switching off UCP protonophoretic function will reemploy the capacity of cell antioxidant systems and consequently they will be no longer capable of preventing the ongoing redox signaling such as retrograde signaling from the mitochondrion. In this way, the UCP switch off may extend the redox signal not only to the cell cytosol but also to ECM. We should note that the descriptions (i) and (ii) refer to the same phenomenon viewed from two different perspectives of interpretation.

Termination of redox signaling by switching on UCP protonophoretic function can be viewed as either (iii) a direct decrease of mitochondrial ROS sources previously substantiating the redox signal by surplus superoxide formation, or, alternatively, (iv) as a release of the extra capacity of cell antioxidant systems, which will terminate the redox signal.

Besides many regulatory factors that can switch on UCP-mediated protonophoretic function as described hereunder, there is a wide variety of effectors of UCP gene expression together with a relatively short half-life of the protein. Thus, for example, a stress character of fast UCP2 protein expression followed by its fast degradation can contribute to redox signaling. However, a description of the vast field of UCP expression and its regulations could be only briefly touched in this review (section V).

For acute regulations, one may predict that the H_2_O_2_-induced activation of iPLA_2_γ (180, 188, 186) represents a terminating factor of the redox signaling in which UCPs participate. Thus, the stimulus initiating the redox signaling can be given in the presence of adequate nascent FAs (see section III.D) by the increase in ATP/AMP or GTP/GMP ratios or decrease in Mg^2+^ (factor decreasing purine nucleotide inhibition of UCPs (199). This would act also in the presence of a UCP anionic substrate for which an active proton symporter exists in IMM. A typical example is pyruvate, which can be expelled in a uniport mode from the matrix by UCP2, whereas its return is ensured by the pyruvate carrier. In this way, the resulting pyruvate cycling (91, 191) would attenuate/terminate redox signal. In contrast, the sudden absence or lowering of pyruvate levels would initiate a redox signal or amplify the ongoing one. Similar speculations might be expected for the anion/Pi antiport mode of UCP2.

Since also a reversible glutathionylation was demonstrated to act as a control switch for UCP2- and UCP3-dependent uncoupling (263), its participation in UCP-involved redox signaling is predicted. Inhibitory glutathionylation should initiate redox signaling, whereas excessive mitochondrial ROS lead to deglutathionylation and hence activation of UCP2, which would terminate redox signaling (266). A threshold should be found, above which signaling turns toward the oxidative stress.

## VII. Noncanonical Roles of Mitochondrial UCPs

### A. Extrusion of organic anions from the matrix by UCP2-mediated antiport

Anion uniport function has long been known for UCP1 (191, 196) and UCP2 (91). The latter was verified recently and an important physiological role has been ascribed to export of organic anions through UCP2 from the matrix in exchange for phosphate (415). When such an export of oxaloacetate (OAA), malate, and aspartate occurs, acetyl-CoA oxidation is diminished, which decreases the substrate pressure, OXPHOS intensity, and at least the complex I I_F_-related superoxide formation. OAA as the minimum accumulated substrate can be thus easily exhausted from the Krebs cycle and UCP2 may replenish this substrate, by catalyzing the OAA export, and make OXPHOS partly dormant, and, consequently, UCP2 promotes higher glucose utilization by aerobic glycolysis (415). However, aspartate transport mediated by UCP2 has been recently excluded in the brain glial compartment (77). Nevertheless, the revealed enrichment of glutamate and its metabolites in brains of UCP2 knockout mice indicates a blocked export of a certain metabolite in the mitochondrial matrix (77).

Also, UCP2-mediated alternation of normal metabolism into the specific cancer metabolism has been reported. Such cancer-specific remodeling is given by the UCP2-mediated rerouting of C4 metabolites that also interferes with information signaling (122). In Figure 11, we predict four modes that could contribute to such rerouting, two models when malate/aspartate carrier within the classic malate–aspartate shuttle is substituted by UCP2 (Fig. 11A, C), the second one when malate/aspartate or 2-oxoglutarate carrier are substituted by UCP2 for export of malate during malate/pyruvate shuttle (Fig. 11B), existing, for example, in pancreatic β-cells (214),^.^and compare them with the previously suggested pyruvate cycling (Fig. 11D) (91, 191).

Physiological roles and effects of redox homeostasis of anion transport UCP2 activities have to be further elucidated. The reconstituted UCP2 exchanged phosphate for preloaded aspartate, malate, OAA, and physiologically irrelevant sulfate and malonate (415). Previously indentified (91) UCP2 substrate pyruvate was not exchanged, but a slight 2-oxoglutarate/phosphate exchange has been detected (415). UCP2 thus would represent an exception to the rule that divalent anions are not translocated by UCPs (191, 196) or these anions had to be in their monovalent forms. Actually, phosphate together with proton(s) directed to the matrix has been considered to be exchanged for the C4 divalent anions such aspartate, malate, and OAA (415). Thus, the export of C4 species would be ensured on the expense of Δ*pH*. If, however, more probable antiport exists *in vivo*, enabling the export of divalent C4 anions for monovalent phosphate, this should directly dissipate Δ*Ψ*_m_.

No data on organic anion transport by UCP3 are available to date. Nevertheless, a recent study concluded that UCP3 activity also affects metabolism beyond FA oxidation, since UCP3 evidently regulates biochemical pathways of amino acid metabolism but also those influencing redox status (7).

### B. Mutual relationships between the FA cycling and the anion transport function

*In vitro* experiments including those studying UCP2 structure (31) always encountered a strong competition of FAs with small anion transport substrates of UCPs in contrast to alkylsulfonates that outcompete FAs. FAs inhibit anion uniport *via* reconstituted UCPs (197, 200), hence *in vivo* effects in the presence of FAs or at ongoing β-oxidation are predicted to be less pronounced than those simulated *in vitro*. As also demonstrated, organic anion cycling may uncouple mitochondrion (91, 191) and hence attenuate Δ*Ψ*_m_-dependent superoxide formation. One may predict that the higher the contribution of FA metabolism in a given cell condition, the lower the contribution of antiport mode for C4/P_i_ will be allowed.

### C. Relationships between uncoupling and mitochondrial calcium transport

**FIG. 13.**
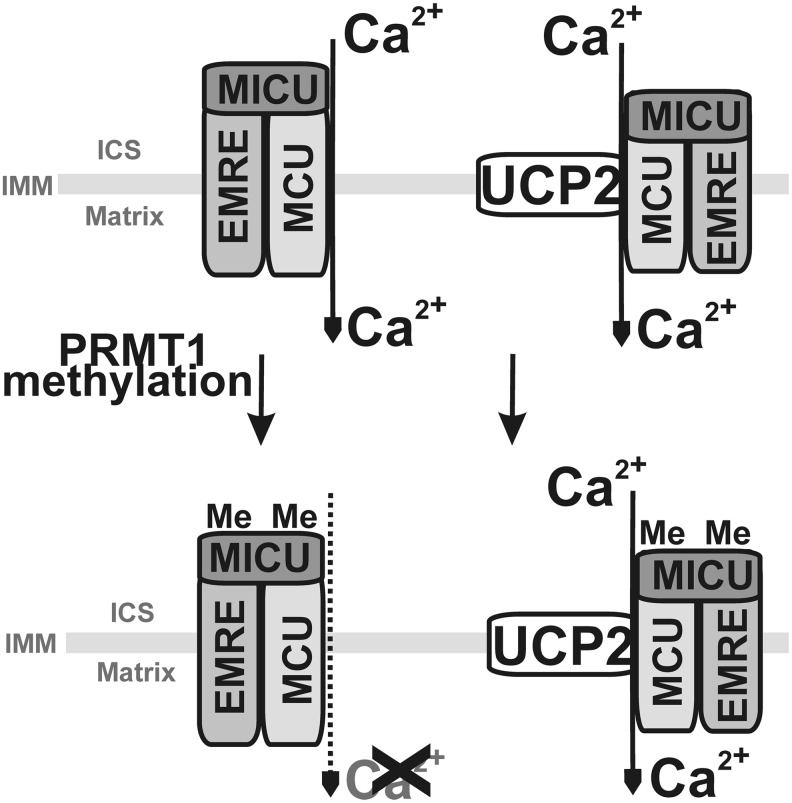
**Calcium transport is affected by UCP2 and UCP3.** Participation of UCP2 (UCP3) in concert with methylation of MICU1 protein controlling MCU in complex with EMRE protein. *Left panels—*in the UCP absence: the protein arginine methyl transferase PRMT1 methylates the MICU1 protein at position 455, thus reducing its sensitivity to Ca^2+^. *Right panels—*in the UCP presence: the sensitivity of the Ca^2+^ uniport becomes unaffected by PRMT1. The scheme was derived from results in Ref. (260). EMRE, essential MCU regulator; MCU, mitochondrial calcium uniporter; MICU1, methylation of mitochondrial Ca^2+^ uptake 1; PRMT1, protein arginine methyl transferase 1.

Another controversial issue was initiated by the observation that UCP2 and UCP3 are fundamental for the regulation of Ca^2+^ levels in mitochondria (400) (Fig. 13). Mitochondria were the first organelles associated with Ca^2+^ handling and the process of mitochondrial Ca^2+^ accumulation has been thoroughly investigated for several decades [progressively reviewed in Refs. (96, 148, 149, 333)]. Thus, mitochondria accumulate Ca^2+^ by a rapid electrophoretic pathway that transports Ca^2+^ into the matrix, driven by the Δ*Ψ*_m_ component of Δ*p* established by the respiratory chain. The pathway was termed “mitochondrial calcium uniporter” (MCU) long before its molecular identity was determined.

In 2007, using overexpression, knockdown, and mutagenesis experiments, Trenker and colleagues demonstrated that UCP2 and UCP3 are elementary for mitochondrial Ca^2+^ sequestration in response to cell stimulation under physiological conditions—observations supported by isolated liver mitochondria of UCP2 knockout mice (400). Because the MCU has been already studied for several decades, these results, which suggested a novel molecular function for UCP2 and UCP3 in the context of mitochondrial Ca^2+^ sequestration, have stimulated both excitement and skepticism in the field.

The role of UCP2/3 in the regulation of mitochondrial Ca^2+^ transport was challenged by a joint report of two independent laboratories, showing that by using mitochondria from four different tissues together with corresponding mitochondria isolated from tissues of UCP2 and UCP3 knockout (UCP3 KO) mice, no direct participation of UCP2 or UCP3 in the mitochondrial Ca^2+^ transport was found (49).

Further evidence has been gradually accumulated in support of the role of UCP2 and UCP3 in the regulation of mitochondrial Ca^2+^ (37, 127, 128, 260, 290, 291, 417–419), although in some reports the role of UCP2 or UCP3 in mitochondrial Ca^2+^ homeostasis was not supported (93, 213). Nevertheless, the contribution of UCP2 and UCP3 to mitochondrial Ca^2+^ uptake was found to be differentially determined by the source of supplied Ca^2+^ (419). Furthermore, by substituting the intermembrane loop 2 (H2) of UCP2 and UCP3 by that of UCP1, these chimeras had no activity in mitochondrial uptake of intracellular released Ca^2+^, while they mimicked the wild-type (wt) proteins by potentiating mitochondrial sequestration of entering Ca^2+^. Mutants of UCP3 at basic residues 168 and 171/172 revealed that distinct sites in the intermembrane loop 2 of UCP3 adjust mitochondrial uptake to high and low Ca^2+^ signals (417).

The molecular identification of proteins involved in the control of Ca^2+^ fluxes across the IMM subsequently followed (27, 95, 171, 213, 269, 323, 339, 362). The progress initiated more detailed studies on the molecular mechanism of UCP2 or UCP3 interaction with the individual components of the mitochondrial Ca^2+^ uptake. The impact of UCP2 on three distinct mitochondrial Ca^2+^ currents found in mitoplasts isolated from HeLa cells was investigated using the patch clamp technique. UCP2 was identified as a selective modulator of just one distinct mitochondrial Ca^2+^ inward current, dependent on the pore-forming mitochondrial MCU protein and the MCU regulator, EMRE (37). In addition, cardiac single-channel activity in mitoplasts of mCa1 current, which is supposed to underlie the MCU in the human and murine heart, suggested that beyond UCP2, UCP3 also exhibits regulatory effects on cardiac mCa1/MCU function (289).

More recently, the molecular mechanism that determines the UCP2 and UCP3 dependency of mitochondrial Ca^2+^ uptake has been further clarified (260). The mitochondrial Ca^2+^ uptake has been shown to be under the control of post-translational protein modification by protein arginine methyl transferase 1 (PRMT1). Namely, the PRMT1 mediates methylation of mitochondrial Ca^2+^ uptake 1 (MICU1), a regulatory subunit of the mitochondrial Ca^2+^ channel macromolecular complex that shields mitochondria from Ca^2+^ overload. Methylation of MICU1 results in a decreased Ca^2+^ sensitivity for protein rearrangement and, thus, a decreased Ca^2+^ uptake. However, such decreased Ca^2+^ sensitivity is not manifested upon interaction of UCP2 or UCP3 with the MCU/MICU multicomplexes (Fig. 13).

Thus UCP2 and UCP3 function as unique regulators of methylated MICU1 that becomes fundamental for mitochondrial Ca^2+^ uniport under conditions of elevated PRMT1 activity (260). Finally, UCP4 was found to regulate Ca^2+^ homeostasis and sensitivity to store depletion-induced apoptosis in neurons (60).

Despite the accumulating evidence of the molecular mechanism of UCP2 and UCP3 interaction with the mitochondrial Ca^2+^ uniporter complex, the established properties of UCP2 and UCP3 to dissipate the Δ*p* and to catalyze transport of FA^−^ anions (179, 182) should also be considered when interpreting their participation in mitochondrial Ca^2+^ transport. The dissipation of Δ*p* strongly affects the steady-state mitochondrial Ca^2+^ levels, decreasing both the Δ*Ψ*_m_-dependent Ca^2+^ uniport uptake and Δ*pH*-dependent Ca^2+^ efflux *via* Ca^2+^/Na^+^ or Ca^2+^/H^+^ antiporters (96). Furthermore, because FA anions can also form ion pairs with Ca^2+^ ions (284), the ability of Ca^2+^ to form complexes with FA anions can hypothetically result in the net UCP2/3-catalyzed Ca^2+^ fluxes under certain experimental conditions.

### D. Involvement of UCPs in mitochondrial network dynamics and cristae morphology

#### 1. Mild uncoupling promotes fission and mitophagy

In intact cells at normal metabolic regimes, mitochondria form a nearly completely connected network of mitochondrial reticulum. Such a completely connected mitochondrial network exists even at skeletal muscle (144) and heart (116, 309). Machinery of proteins governing the fission (leading to a fragmentation of tubules into small spheroids) and fusion (rejoining fragments into the major network) allows a fine dynamic balance between fission and fusion so that they are in near equilibrium. Prevailing fission is employed for mitochondrial-specific autophagy (termed mitophagy) of fragments exhibiting low Δ*p* (Δ*Ψ*_m_) (404), typically due to capturing fragments containing mutant mtDNA or predominant population of oxidized and nonfunctional proteins (both leading to the inevitable Δ*Ψ*_m_ drop).

**FIG. 14.**
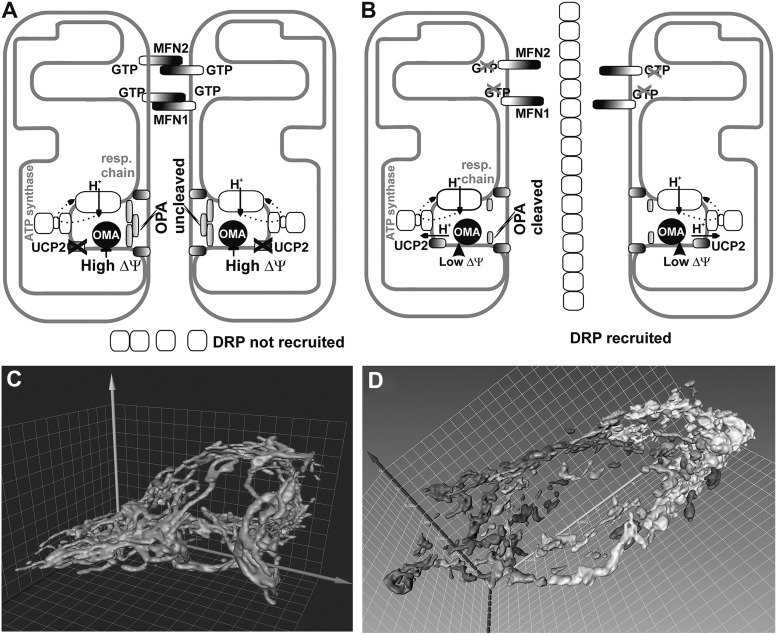
**UCP2 role in promoting fragmentation of mitochondrial network. (A)** Completely continuous mitochondrial network reticulum is established when fission/fragmentation is balanced by fusion and DRP1 protein is not fully recruited toward the outer membrane. The UCP2 blockage helps to set the balance. **(B)** Switching on the UCP2-mediated uncoupling may set ΔΨ_m_ below a threshold required for activation of OMA1-mediated cleavage of profusion protein OPA1, and by enabling lower GTP for mitofusin 1 (“MFN1”) and mitofusin 2 GTP-ases (“MFN2”). **(C**, **D)** 3D 4Pi microscopic images of insulinoma INS1E cell mitochondrial network at continuous or fragmented morphology [measurements similar to those in Ref. (330)]. Grids: 1 μm.

This housekeeping cleaning function in the cell thus can be accelerated or promoted by the enhanced mild uncoupling. Uncoupling is a key stimulus among numerous insults, resulting in the prevailed fission of mitochondrial network. The mechanism is based on the strong dependency of OMA1 on Δ*Ψ*_m_ (cleavage is activated upon its drop, (449) and of other proteases (216, 234, 416), all cleaving OPA1, a major profusion protein (206, 259, 322, 361). During fission, DRP1, the GTPase fundamental component of mitochondrial fission machinery, is recruited toward the OMM, depending on the local cellular energetics and information signaling (Fig. 14) (221, 241, 441). The local cytosolic GTP concentrations govern fission by multimerization of DRP1, resulting in formation of rings around dividing mitochondrial tubules at the so-called “constriction points.” Fission cannot prevail, since the profusion mitodynamins such as mitofusins MFN1 and MFN2 (366) are equally affected by GTP. However, fission will prevail when profusion long OPA1 isoforms are cleaved.

Thus, autophagy induction by palmitic acid was dependent on UCP2 in hepatocytes (257). Endogenous UCP2 was reported to modulate endothelial mitochondrial network morphology that regulates endothelial cell function (371). Increased fragmentation has been observed under different conditions, elevating UCPs or UCP1 functionality (429). Beneficial effects of UCPs on the oxidative status can, therefore, be promoted by this indirect mechanism, when higher fission occurrence leads to a higher frequency of checking of fragments with the oxidized proteins (and/or with mutant mtDNA that itself leads to enhance oxidative stress within a given fragment). As a result, a higher extent of crippled mitochondrial fragments is eliminated by mitophagy, hence less oxidized material/mutant mtDNA remains within the cell and thus the oxidative stress is diminished as well as the resulting superoxide formation in the more intact remaining network.

UCP2 has been recently indicated to be critical for mitochondrial fragmentation in neurons of ventromedial nucleus of the hypothalamus, which is an inherent part of glucose sensing for these neurons (395). UCP2 participates in the glucose-stimulated phosphorylation of DRP1 fission protein, recruited to the mitochondrial tubules, which are subsequently fragmented. The involvement of UCP2 uncoupling-related antioxidant action (186) has been evidenced by observation of ROS increase after glucose administration in UCP2-ablated mice (395). Since mechanism of hypothalamic glucose sensing is similar to pancreatic β-cell glucose sensing (230), see section VIII.E), one might predict similar fission involvement in pancreatic β-cells.

#### 2. Mild uncoupling reshapes cristae

Upon a complete fragmentation of mitochondrial network, cristae within small spheroid fragments are largely lost and the fragments possess a distinct morphology with parts of the OMM forming interior spheroids within a spheroid, thus greatly differing from the intact and even apoptotic morphology (102). Often the fragments engulfed a portion of the cytosol. At lower resolution, such engulfment is reflected by apparent matrix space toroids (330). Thus, even a mild uncoupling may have a profound effect on cristae morphology and so represents an independent regulatory entity with less efficient ATP synthesis (329).

## VIII. Involvement of UCPs in Redox Homeostasis and Redox Regulations

### A. Regulation of redox-sensitive kinase signaling by UCPs

Mitochondria-derived oxidants became recognized as important signaling molecules that communicate between mitochondria and the rest of the cell under physiological conditions (61, 427). UCP2 has already been reported to participate in cellular redox signaling originating from the mitochondrion (118, 186, 264). Also, any redox signaling can be terminated by a sudden increase of the redox buffering capacity within the mitochondrial microenvironment.

Mitochondria play a key role in the regulation of physiological cell function through redox cell signaling (150), including cardioprotective signaling and prevention of programmed cell death (133). H_2_O_2_ became generally accepted as the predominant intracellular redox-signaling molecule, being able to oxidize catalytic cysteine thiol groups of protein tyrosine phosphatases and numerous protein kinases (79, 343). In addition, there is increasing evidence pointing to the role of the protein kinase C (PKC) family of isoenzymes in transducing H_2_O_2_-induced signaling in a wide variety of physiological and pathophysiological processes (80, 132, 143, 145, 178, 231, 359).

Based on the mentioned hypothetical assumptions for UCP participation in redox signaling (section VI.C), we may predict that an increase within the mitochondrial ICS compartment (or within the overall intermembrane space, *i.e.*, diffused from the cytosol) for ATP_ICS_/AMP_ICS_ or GTP_ICS_/GMP_ICS_ ratios or decrease in Mg_ICS_^2+^ will initiate signaling by the redox-activated kinases by acute inhibition of UCP-mediated uncoupling. In contrast, increased pyruvate cycling at higher pyruvate levels or increased FAs available and upon redox activation of iPLA_2_γ in the simultaneous (or sole) decreased ATP_ICS_/AMP_ICS_ or GTP_ICS_/GMP_ICS_ or increased Mg_ICS_^2+^ will terminate signaling by the redox-activated kinases. Even though such a function for UCPs remains largely unexplored, a recent line of evidence suggests a role for UCPs in the regulation of redox-sensitive protein phosphatases and kinases (Figs. 8A and 12).

Emre *et al.* (118) found that UCP2 activation switches off redox signaling in macrophages, substantiating signal amplification loop that enhances mitogen-activated protein kinase (MAPK) pathway. Lipopolysaccharide normally downregulates UCP2 through the c-Jun N-terminal kinase and p38 pathways and this was shown to increase mitochondrial ROS production. This further stimulated MAPK and thus acted in the signal amplification loop potentiating MAPK pathway activation.

Consistent with this, UCP2-deficient macrophages exhibited an enhanced inflammatory state (22, 349) characterized also by the increased nitric oxide production and elevated migration ability (118). Another work examined the UCP2 role in mice erythropoiesis, where UCP2 facilitates heme synthesis and iron metabolism by attenuating ROS production. Analysis of progenitor cells from bone marrow and fetal liver both *in vitro* and *in vivo* revealed that UCP2 deficiency results in a significant decrease in cell proliferation at the erythropoietin-dependent phase of erythropoiesis. This was accompanied by a reduction in the phosphorylated form of MAPK/ERK (117).

An interesting role of UCP2 has been found in association with *Leishmania* parasites impairing the generation of ROS, which is a major host defense mechanism against any invading pathogen. It has been demonstrated that *Leishmania donovani* infection is associated with strong upregulation of UCP2. In contrast, functional knockdown of macrophage UCP2 by siRNA-mediated silencing was associated with increased mitochondrial ROS generation, lower parasite survival, and induction of marked proinflammatory cytokine response. Induction of proinflammatory cytokine response in UCP2 knocked down cells was a direct consequence of p38 and ERK1/2 MAPK activation, which resulted from oxidant-mediated inhibition of protein tyrosine phosphatases. In addition, *in vivo* silencing of UCP2 resulted in decreased Src homology 2 domain-containing tyrosine phosphatase 1 and protein tyrosine phosphatase-1B activity, and host-protective proinflammatory cytokine response resulting in effective parasite clearance (26).

Using an uncoupling agent FCCP, it has been demonstrated that mild mitochondrial uncoupling prevented premature senescence in human dermal fibroblasts by attenuating the alteration in redox state and suppressing redox-dependent c-Jun N-terminal kinase signaling cascade (69). Employing a UCP3 transgene targeted to the basal epidermis, it has been shown that forced mitochondrial uncoupling inhibits skin carcinogenesis by blocking the activation of protein kinase B (Akt). Similarly, Akt activation is markedly inhibited in UCP3 overexpressing primary human keratinocytes (304).

Another link between UCP function and regulation of cytoprotective signaling cascade was indicated by studies showing that UCP3 mediates the cardioprotection of H_2_O_2_-induced preconditioning by preserving the mitochondrial function through inhibiting the mitochondrial permeability transition pore opening, and that UCP3 overexpression augmented Akt and GSK-3β phosphorylation signaling pathways, indicating that the PI3K/Akt/GSK-3β signaling pathway is partially involved in the UCP3-afforded cardioprotection (66).

### B. Regulation of insulin secretion

Applying the hypothetical predictions of activation of UCP2-mediated uncoupling to physiology of pancreatic β-cells, one may envisage the specific role of UCP2 in these glucose-sensing cells. An increase in ATP_ICS_/AMP_ICS_ or GTP_ICS_/GMP_ICS_ (or decrease in Mg_ICS_^2+^) may initiate certain redox signaling. Note that upon GSIS, ATP is indeed elevated due to more intensive OXPHOS (ATP synthesis). In contrast, the increased pyruvate cycling at higher pyruvate levels (which would accumulate after termination of glucose intake by β-cells) and ATP/AMP or GTP/GMP would terminate the putative redox signaling. Moreover, the suggested C4 anion exchange for phosphate incoming with a proton to the matrix (415) may participate in redox shuttles transferring redox equivalents into the form of cytosolic NADPH (334). For example, the resulting malate export mediated by UCP2 would theoretically ensure this (Fig. 11C), by participating in so-called pyruvate/malate shuttle (214).

Redox-stimulated insulin secretion was demonstrated in β-cells and represents a portion of the stimulated insulin release that can be regulated by UCP2 deficiency or inhibition (180, 186). Moreover, the FA-stimulated insulin secretion exists in pancreatic β-cells, in which iPLA_2_γ and UCP2 directly participate by amplifying the initial signal of administered exogenous FAs (186).

Recent evidence suggests that iPLA_2_γ is directly activated by H_2_O_2_. In addition, physiologically relevant concentrations of exogenous palmitic acid were not able or sufficient to directly activate the G-protein-coupled receptor-40 (GPR40), but instead were metabolized by mitochondrial β-oxidation that produces superoxide and subsequent H_2_O_2_, resulting in the activation of iPLA_2_γ. The iPLA_2_γ catalyzed cleavage of FAs not only switches on the UCP2 function and concomitant antioxidant protection but is also able to release FAs that diffuse toward the plasma membrane (186). These results indicate that the key role is played by the intrinsic FAs that activate GPR40 receptors and *via* FA/glycerolipid cycle stimulate insulin secretion (334). This example represents a relay of the initial weak FA-mediated signaling amplified *via* redox signaling to the mitochondrial FA-mediated signaling. Hypothetically, such a relay may exist in other cell types, at least those wherein iPLA_2_γ is sufficiently expressed, such as heart, brain, lung, kidney, and other tissues.

Also, a reversible S-glutathionylation inhibiting UCP2- and UCP3-dependent uncoupling (267) was reported to enhance GSIS and, conversely, increase in mitochondrial ROS that deglutathionylates UCP2 and initiates UCP2-impeded GSIS (264). An increase in mitochondrial matrix oxidants reversed the S-glutathionylation and increased the UCP2-catalyzed activity, leading to impeded GSIS. Elevated glucose metabolism also decreased the total amount of cellular glutathionylated proteins and increased the cellular GSH redox ratio (GSH/GSSG), indicating that the glutathionylation status of UCP2 contributes to the regulation of GSIS (264).

The profound influence of UCP2 on redox homeostasis in pancreatic β-cells has been previously reported (192). UCP2 was found to decrease the yield of ATP from glucose (310, 445). The addition of natural aglycone genipin, a putative UCP2 inhibitor (438), caused a Δ*Ψ*_m_ increase in wt pancreatic islets but not in UCP2 knockout islets (446). UCP2 overexpression in INS-1 β-cell model attenuated IL1β-induced ROS formation (335). A mild uncoupling in mitochondria isolated from INS-1E cells was linked to UCP2, while accounting for up to 30% of H^+^ leak (5, 6). In contrast, Galetti *et al.* (130) could not demonstrate any effect of UCP2 overexpression on mitochondrial coupling in INS-1 cells, neither after oleate addition.

An antioxidant role of UCP2 in pancreatic β-cells has been evidenced for UCP2 knockout mice of three highly congenic strain backgrounds, all exhibiting oxidative stress (decreased ratios of reduced-to-oxidized GSH in blood or tissues), elevated levels of antioxidant enzymes, and increased nitrotyrosine content in their islets (327). Pancreatic β-cells from UCP2-deficient mice exhibited chronically higher ROS than those from wt mice (246). Also, mice with selective knockout of UCP2 in pancreatic β-cells exhibited increased glucose-induced Δ*Ψ*_m_ and elevated intracellular ROS (347). UCP2 has also been indicated in normal α-cell glucose sensing and the maintenance of euglycemia (9).

Impaired redox signaling and antioxidant protection manifested by UCPs can contribute to type 2 diabetes development. Concerning the etiology branch of disrupted pancreatic β-cell biogenesis and function, the excessive UCP2-mediated uncoupling may prevent the correct responses on glucose, hence impair also the beneficial effects of autocrine insulin and other hormones (incretins). The disrupted autocrine secretion and housekeeping and biogenesis of β-cells stimulate secretion of IL-1β or other cytokines or chemokines and attract immune cells (344). As a result, nearly systemic inflammation spreads causing the impaired glucose tolerance at the first stage and the insulin resistance in the progressed disease.

Alternatively, shifted balance due to the nonfunctional UCP2 in white adipose tissue may lead to insulin resistance beginning with inflammation of the adipocyte origin. It is well established that a shift from an anti-inflammatory M2 macrophage state toward a proinflammatory M1 macrophage state determines the obesity inflammatory phenotype of white adipose tissue, which may spread systemically to cause insulin resistance (278). Also, the lack of UCP-mediated antioxidant protection on one hand (177, 286), or excessive uncoupling on the other hand (41), participates in the amplification of oxidative stress in peripheral tissues during type 2 diabetes development (301).

### C. Redox regulations in endothelial cells

A typical example of UCP2 being involved in signaling cascades has been found for downregulation of cyclooxygenase-2 expression due to activation of the GLP1 receptor AMPK cascade in endothelial cells of mouse aortae (256). Also, adenovirus-mediated UCP2 overexpression led to a significant increase in endothelial nitric oxide synthase and decrease in endothelin-1 mRNA expression in human aortic endothelial cells. Moreover, UCP2 inhibited the increase in ROS production and apoptosis, which suggests that UCP2 functions as a physiologic downregulator of ROS generation and redox signaling in endothelial cells (242). Specific signaling of ghrelin inhibiting inflammatory response to oxidized low-density lipoprotein was found to increase UCP2 and hence terminate redox signaling (353).

Further studies attempted to investigate how the endogenous UCP2 modulates endothelial mitochondrial network morphology that regulates endothelial cell function (371). Upregulation of UCP2 was critical for controlling mitochondrial membrane potential and superoxide production. In the absence of UCP2, endothelial growth stimulation provoked mitochondrial network fragmentation and premature senescence *via* a mechanism involving redox-mediated p53 activation. Mitochondria thus preserve normal network integrity and impact cell phenotype *via* regulation of UCP2 (371). Further studies described a functional characteristic and an antioxidant role for UCP2 in endothelial cells and isolated mitochondria. They indicated that endothelial UCP2 may function as a sensor and negative regulator of mitochondrial ROS production in response to hyperglycemia (235, 236). Thus, for example, glucocorticoids diminish mitochondrial superoxide formation by upregulation of UCP2 in endothelial cells upon hyperglycemia (140).

UCP2 acting as an adaptive antioxidant defense factor protected against mitochondrial ROS-induced endothelial dysfunction in atherosclerosis (434). UCP2 is upregulated *via* the activation of transient receptor potential vanilloid 1 (TRPV1), leading to ameliorated coronary dysfunction and prolonged lifespan of the atherosclerotic mice. The likely promoting of redox signals due to the loss of endothelial UCP2 induces PTEN-induced putative kinase-1 after signaling, leading to mitophagy during intermittent hypoxia at developing pulmonary hypertension (156).

### D. Redox regulations of cell cycle

Mitochondrial signaling cascades have been implicated in the activation of programmed cell death and the control of cell proliferation (358), and the role of UCP2 in the regulation of the cell cycle has recently been indicated. Haines and Li (151) tested the hypothesis that UCP2 is neuroprotective by suppressing innate inflammation and regulating cell cycle mediators. PCR gene arrays and protein arrays were used to determine mechanisms of damage and protection after transient focal ischemia. The results showed that ischemia increased the expression of inflammatory genes and suppressed the expression of antiapoptotic and cell cycle genes. UCP2 also increased the expression of cell cycle genes and protein levels of phospho-AKT, PKC, and MEK after ischemia. It was concluded that the neuroprotective effects of UCP2 against ischemic brain injury are associated with inhibition of proinflammatory cytokines and activation of cell survival factors (151).

The effect of UCP2 expression on cell proliferation and viability was investigated using UCP2-transfected Hepa 1–6 cells. Flow cytometry analysis indicated that UCP2-transfected cells were less proliferative than nontransfected controls, with most cells blocked at the G1 phase. This effect of UCP2 was augmented by treatment with genistein, a tyrosine kinase inhibitor, which by itself did not affect cell proliferation on control hepatocytes. Examination of cell viability in UCP2-transfected cells revealed that UCP2 significantly increased cell death. However, characteristics of apoptosis were absent in UCP2-transfected Hepa 1–6 cells. These results indicate that UCP2 induces cell cycle arrest at G1 phase and causes nonapoptotic cell death, suggesting that UCP2 may act as a powerful influence on hepatic regeneration and cell death in the steatotic liver (314).

### E. UCP involvement in the central regulation of metabolism

A shift toward glucose utilization in brown adipose tissue impairing the cold-induced nonshivering thermogenesis has been reported upon loss of UCP2 (55). It is not known whether UCP2 influence is manifested at the brown adipocytes level or at a central level. The regulations of the central level are described hereunder.

Involvement of UCPs in hypothalamus, which is a key controlling center for energy metabolism and homeostasis, has been surprisingly found to mimic similar principles of UCP involvement in other cell types. Hypothalamic anorexigenic neurons express proopiomelanocortin, whereas orexigenic neurons express neuropeptide Y and agouti-related peptide. FA-mediated redox signaling may control release of these neuropeptides. Thus, the UCP2 ablation has been reported to diminish fasting- as well as ghrelin-induced food intake due to the resulting impaired orexigenic neurons by the elevated ROS (13, 78).

Elevated ROS by FA β-oxidation were involved in the implicated antioxidant action (13). Note that such mechanism might be similar to that recently described for pancreatic β-cells (186). In proopiomelanocortin neurons, an ROS scavenger as well as UCP2 activation decreased their proopiomelanocortin secretion, which is specifically important at elevated glucose and leptin levels and substantiates the phenomenon of the so-called leptin-resistance (101). The direct UCP2 involvement in glucose sensing of glucose-excited proopiomelanocortin neurons was reported to be identical to glucose sensing of pancreatic β-cells (230, 317).

Recently, UCP2 participation in glucose sensing in neurons of ventromedial nucleus of the hypothalamus has been related to fragmentation/fission of the perinuclear mitochondrial network (395). By yet unspecified mechanism, UCP2 promotes the glucose-stimulated phosphorylation of DRP1 fission protein, besides its general uncoupling-related antioxidant action (395).

### F. UCP involvement in cardioprotection

**FIG. 15.**
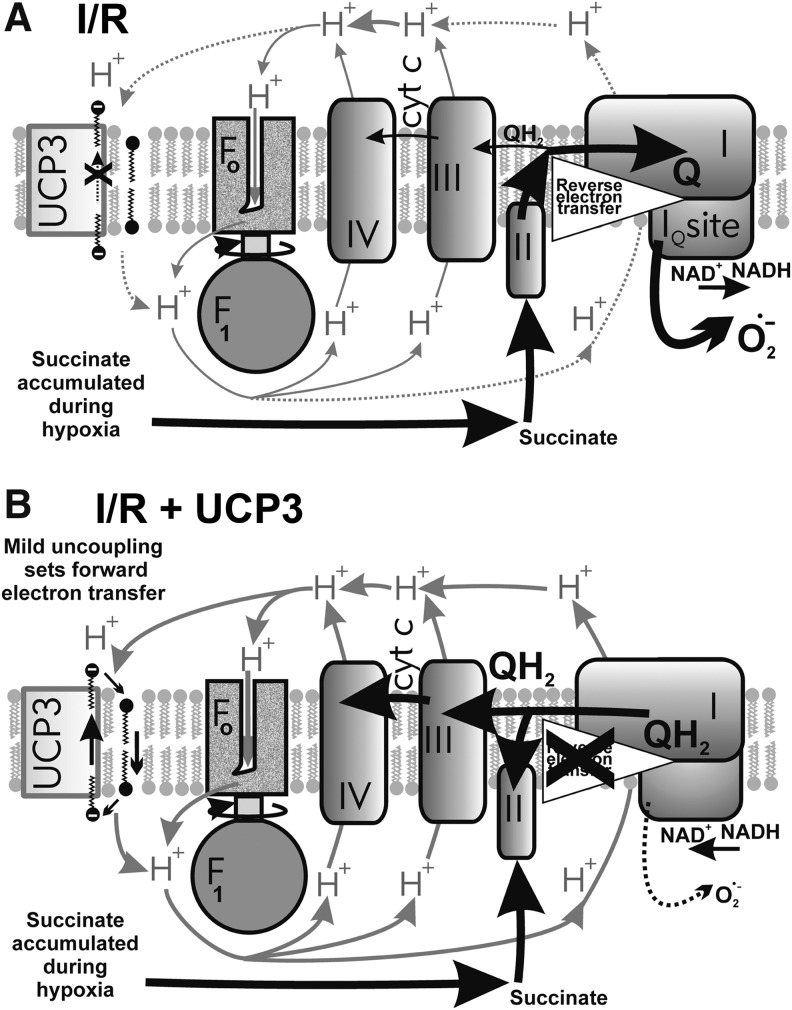
**UCP3 in cardioprotection against I/R injury. (A)** Superoxide formed at instant reperfusion: high succinate accumulated in ischemic period is metabolized fast so to promote reverse electron transfer from succinate dehydrogenase (complex “II”) toward the I_Q_ site of complex I, where a high superoxide formation occurs (73, 74, 103). **(B)** UCP3-mediated mild uncoupling is able to redirect electron transfer to the forward one: similarly, as depicted in Figure 3A, higher rates of respiration and proton pumping promoted by mild uncoupling attenuate superoxide formation at complex I I_Q_ site. Derived on the basis of results in Refs. (313) and (325, 392). *Thicker arrows* indicate higher fluxes and *dotted lines* illustrate the absence of fluxes. Orientation: *bottom parts* represent the matrix; *upper parts* represent the ICS. I/R, ischemia/reperfusion.

UCP2 and UCP3 are expressed at low amounts in the heart, where their regulation can contribute to uncoupling and hence attenuation of mitochondrial superoxide formation (52, 450), leading to cardioprotection (Fig. 15). Early reports have shown that delayed ischemic preconditioning (IPC), which promotes cardioprotection *via* genomic reprogramming, upregulates UCP2 and UCP3 after the upregulation of PPARγ coactivator 1α (277, 386). Subsequent examination of the molecular and biochemical regulation of cardiac UCP2 and UCP3 in delayed preconditioning showed that the regulatory events are ROS inducible and appear to attenuate the subsequent anoxia-reoxygenation-mediated mitochondrial ROS production (276).

Using adenoviral vector containing human UCP2, the effect of UCP2 overexpression on the mitochondrial death pathway induced by oxidative stress was investigated in cultured neonatal cardiomyocytes (392). Thus, UCP2 overexpression suppresses markers of cell death and prevents the loss of IMM potential induced by H_2_O_2_, which is a critical early event in cell death. UCP2 overexpression also prevents Ca^2+^ overload and decreases the production of ROS, thus supporting UCP2-dependent mechanism of cardioprotection (276, 392).

The nonischemic doxorubicin-induced heart failure is associated with decreases in UCP2 and UCP3 protein expression, which is accompanied with increased oxidative stress (51). On the contrary, UCP3 is upregulated in the myocardium of chronically infarcted rat heart, which is associated with high circulating FFA concentrations, mitochondrial uncoupling, and decreased cardiac efficiency (292).

The effects of loss of cardiac UCP3 function following *ex vivo* and *in vivo* models of ischemia/reperfusion (I/R) injury and IPC were investigated using UCP3 KO and wt mice by Ozcan *et al.* (313). The isolated perfused hearts of UCP3 KO mice were found to have poorer recovery of left ventricular function than wt hearts under I/R conditions. *In vivo* occlusion of the left coronary artery resulted in twofold larger infarcts in UCP3 KO mice than in wt mice. UCP3 KO mice generated more ROS than wt mice during I/R and the protection by IPC was abolished in UCP3 KO mice (313). Thus, UCP3 plays as essential role in the tolerance to I/R injury in the heart and plays a significant role in IPC (313).

The mechanism of UCP3-mediated cardioprotection was further investigated using H_2_O_2_-induced preconditioning before the I/R injury (66), which indicated that UCP3 mediates cardioprotection by preserving the mitochondrial function through inhibiting mitochondrial permeability transition pore opening *via* the interaction with adenine nucleotide translocase and the PI3K/Akt pathway (66).

Concerning the cardiac remodeling in the early stages of heart pathology, a compensation is developed as a slightly increased FA β-oxidation, followed by OXPHOS decline as glucose uptake and glycolysis are upregulated during the pathology progression (131, 229, 249), specifically in obesity (40). At pathogenesis, high UCP2 levels with simultaneously downregulated mitochondrial pyruvate carrier are able to promote a shift from pyruvate to FA oxidation with a parallel establishment of higher aerobic glycolysis (*i.e.*, without OXPHOS) (274). Note that similar shifts are common to be UCP2 mediated in cancerogenesis and in immune cells (see sections VIII.H and VIII.I).

UCP2 has also been shown to participate in cardiac Ca^2+^ handling, influencing susceptibility for Ca^2+^-mediated arrhythmias (240). In addition, cytoprotection by UCP2 attenuation of apoptosis in tubular epithelial cells has been reported in case of renal ischemia/reperfusion injury (452). In general, UCP-mediated dissipation of the protonmotive force and the generation of ROS from the electron transport chain are linked to each other, and there is accumulating evidence pointing to a crucial role of UCPs in the pathogenesis of cardiovascular disease, including cell survival during hypoxia and modulating infarct size in the ischemic heart (67, 285, 292, 325, 360, 402).

### G. UCP involvement in brain and neuroprotection

The role of UCP2 has been indicated even in higher brain functions, neuronal plasticity, and network oscillation (160). Protection in traumatic brain injury by UCP2 has also been reported (303). For example, UCP2 influences retinal ganglion cell number and survival (25). Likewise, hypoxic synaptic remodeling in the cortex and hippocampus was reported to be controlled by UCP2 (407). It has been reported that UCP2 is mostly expressed in glia, whereas the major UCP in neurons is UCP4 (341, 355). This controversy has to be solved for each of morphology or neuronal types.

Since activation of microglias is a hallmark of neuroinflammation, a UCP2 role is suggested in neuroinflammatory and neurodegenerative processes (157). Thus microglia with silenced UCP2 treated with lipopolysaccharide exhibited an elevated inflammatory response, namely higher nitric oxide and interleukin-6 formation (94), suggesting the release of redox signaling promoting inflammation. Glial neuronal interrelationships are undoubtedly involved in the phenomena such as amelioration of apoptotic neuronal cell death in the hippocampus following *status epilepticus*, where UCP2 was found protective (75).

UCP4 has been related to promotion of astrocyte survival (324). The UCP4-mediated neuronal protection has been linked to the NF-κB c-Rel prosurvival pathway (165, 166, 168). Thus, in neuroblastoma, UCP4 was able to increase ATP supply (168). Genetic variants of UCP4 predetermine distinct susceptibility to late-onset Alzheimer's disease (287, 393). The involvement in the Ca^2+^ homeostasis was also reported for UCP4 (60). Also UCP5 has been reported to be neuroprotective (167, 239).

Retrograde and antiretrograde apparent transport of fragmented mitochondria in neuronal axons belongs to an inherent part of their physiology. Longitudinal waves of appearance/disappearance of small mitochondria at the particular axon long coordinate most probably result from permanent fusion/fission cycles that due to GTP-ase character of the involved mitodynamin proteins and OPA1 isoform/cleavage maintenance depend on Δ*p* in these mitochondrial fragments (329) and theoretically also on uncoupling. This has indeed been reported experimentally.

The “movement” resulting from cyclic fragmentation/fission of axonal mitochondria has been described as spontaneous “contractions,” which were concomitant to biosensor-monitored redox changes (47). “Contractions” were amplified at increased neuronal activity and were followed by respiratory chain-related Δ*Ψ* declines, ascribed to uncoupling by UCPs due to the observed sensitivity to genipin (47). The affected mitochondrial fragments exhibited redox state shifted to more oxidant, while mitochondrial antioxidant MitoQ or MnSOD overexpression reduced “contraction” frequency. Upon axotomy, the resulting prooxidant state spread along the axon length (47). Thus, a prooxidant state has been pointed out as a stressor initiating the morphology changes that subsequently led to UCP2-mediated Δ*Ψ* (Δ*p*) declines, transient prevention of elevated ROS formation, which led to transient ROS burst suppression and overall to cyclic changes.

Requirement of neuroprotecting role of UCP2 has already been documented as a prevention of neuronal death and brain dysfunction after stroke and brain trauma (275) and upon UCP2 downregulation by antisense oligonucleotides, which resulted in impaired learning and memory of the mice (421). UCP2 expression in astrocytes was linked to the increased survival of dopaminergic neurons upon Parkinson disease (258). UCP2 overexpression served by antioxidant protection even when complex I-related oxidative stress has been evoked in nigral dopaminergic cells and prevented the cell loss (11).

In synergy, the inhibition of the FA binding protein-4 and activated UCP2 expression elevate redox state and subsequently attenuate palmitic acid-induced proinflammatory response (111). Active UCP2 terminates redox signaling, modulating the nod-like receptor protein 3 inflammasome in astrocytes (110, 258, 388). The signaling *via* NF-κB and thioredoxin-interacting protein (TXNIP) otherwise activates inflammasome (110), thus contributing to depression. During embryonic neurogenesis, active UCP2 terminates redox signaling in the developing neocortex, which otherwise leads to ubiquitination and degradation of transcription factor Yap (211).

### H. UCP involvement in cancerogenesis

A notion that UCP2 is a stress-induced protein is valid also for cancer cells (18, 42). Thus, in most malignant cells as in early developing colorectal cancer (84), nonsmall cell lung cancer (308), leukemia (326), and pancreatic cancer UCP2 levels are elevated (108, 251). Speculations have been reported on both the cancer promoting and protecting role of UCPs, particularly UCP2. Inhibition of UCP2 with genipin was suggested to sensitize drug-resistant cancer cells to cytotoxic agents (261). The promoting mechanisms may involve the prevention of the proper redox regulations at sufficient UCP2 overexpression on one hand, and the promotion by the oxaloacetate (aspartate or malate) export from the matrix, leading to its depletion and facilitation of aerobic glycolysis (415), on the other hand.

**FIG. 16.**
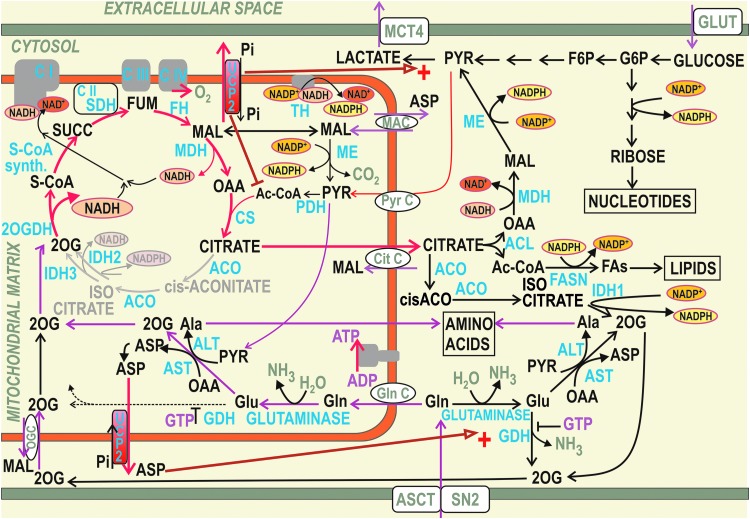
**UCP2 promoting tumorigenesis.** Scheme of major cancer cell pathways including glycolysis, glutaminolysis, and oxidative phosphorylation, emphasizing synthesis and utilization of NADH/NAD^+^ and NADPH/NADP^+^ together with UCP2-promoted glutaminolysis. The latter occurs probably by UCP2-mediated enhancement of the aspartate efflux from the matrix, leading to suppression of glycolytic influx to the Krebs cycle by export of malate, oxaloacetae (not shown), or aspartate from the matrix. Major metabolic fluxes and their absence (*fading symbols and arrows*) or decrease (*smaller symbols and thinner arrows*) are depicted. In cancer cells, a Krebs cycle segment is largely unused that includes aconitase (“ACO”) and isocitrate dehydrogenase 3 (“IDH3”), consequently supplying less NADH for mitochondrial complex I (“CI”) of the respiratory chain (“CIII,”“CIV”), which includes also succinate dehydrogenase (“CII SDH”) of the Krebs cycle. Glutaminolytic pathway (*purple arrows*) supplies 2-oxoglutarate (2OG) for 2OG dehydrogenase (“2OGDH”), thus allowing pyruvate to be metabolized by mitochondria, besides for intensive lactate production. Pyruvate dehydrogenase (“PDH”) is nearly often completely inhibited by phosphorylation in cancer. Dependent on metabolic shuttles, malate (“MAL”) is either imported into the matrix or exported out. The malate export or diversion from the Krebs cycle allows malic enzyme (“ME”) reaction producing NADPH. The malate diversion efficiently leads to a lower NADH production by malate dehydrogenase (“MDH”). At sufficient Δ*p* created by the respiratory chain proton pumping, also transhydrogenase (“TH”) synthesizes NADPH at the expense of NADH. Glutamate dehydrogenase (“GDH”) reaction, which also produces NADPH, is largely inhibited in glutaminolytic cancer cells by GTP. ACL, ATP citrate lyase; ASCT, neutral amino acid exchanger; ASP, aspartate; Cit C, mitochondrial citrate carrier; CS, citrate synthase; FH, fumarate hydratase; FASN, fatty acid synthetase; Gln C, mitochondrial glutamine carrier; GLUT, glucose transporter; MAC, mitochondrial malate aspartate carrier; MCT4, lactate transporter; OAA, oxaloacetate; OGC, oxoglutarate carrier; Pyr C, mitochondrial pyruvate carrier; S-CoA, succinyl coenzyme A; SN2, glutamine transporter. Scheme was drawn according to Refs. (415) and (378).

In the latter case, UCP2 does not act *via* a mild uncoupling but by rerouting the cancer-specific metabolic pathways and/or redox shuttles between mitochondria and cytosol to which it can amend a new transport mode (Figs. 9 and 16). Thus the UCP2 overexpression led to the increased signaling from the master energy-regulating kinase, adenosine monophosphate-activated protein kinase, and HIF dowregulation, all induced reportedly by the introduction of the new C4 anion export mechanism (121, 122).

**FIG. 17.**
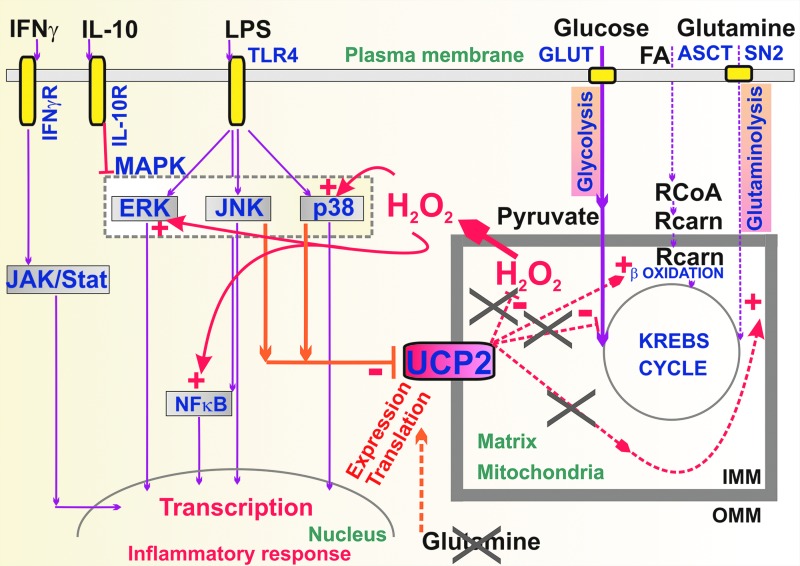
**UCP2 roles in macrophage activation.** UCP2 is highly expressed in resting macrophage. After LPS stimulation, glucose oxidation increases on the expense of glutaminolysis, due to MAPK pathway/mediated downregulation of UCP2 expression. This is communicated by p38 and JNK information signaling pathways leading to suppression of UCP2 expression. Low glutamine levels stop promoting UCP2 mRNA translation. Resulting higher mitochondrial H_2_O_2_ release due to blockage of the UCP2-mediated antioxidant action further stimulate the ERK and NFκB pathways providing the more strengthening downregulation of UCP2 expression. The scheme was modified from Ref. (119). TLR, toll-like receptor. *Arrows*, ongoing regulations or fluxes; *dotted arrows*, slow or inhibited regulations or fluxes; *dotted boxes*.

The tumorigenesis promoting effects of UCP2 or other UCPs are generally based on shifting balance between the aerobic glycolysis (Warburg phenotype) and OXPHOS toward the aerobic glycolysis (Fig. 17) (21). Redox signaling toward gene expression of UCPs themselves may play a significant role in attenuating the elevated ROS, which subsequently cause establishment of higher proportion of aerobic glycolysis.

Thus in skin of MnSOD-heterozygous knockout (Sod2^+/−^) mice, the elevated matrix ROS did induce the PPAR-α activation and increased expression of UCP1, UCP2, and UCP3 (436), suggesting a concomitant protection against the continued production of elevated superoxide. Moreover, PI3K/Akt/mTOR pathway was activated, promoting a higher proportion of aerobic glycolysis *versus* the complete OXPHOS. UCP ablation led to suppression of this pathway. UCP2 ablation also significantly reduced the formation of both benign (papilloma) and malignant (squamous cell carcinoma) tumors (252). Likewise, the ^18^F-fluorodeoxyglucose uptake has been diminished by the putative UCP2 inhibitor genipin, indicating the UCP2 role in a shift toward the Warburg phenotype (70).

Note that a similar mechanism is executed in immune cells (see next section, VIII.I) and in the differentiation of human pluripotent stem cells, where proliferation is related to UCP2-promoted shift toward aerobic glycolysis. With early differentiation, however, when UCP2 is repressed, pluripotent stem cell proliferation becomes retarded due to the decreased aerobic glycolysis and maintained or increased OXPHOS oxidation (448).

The protecting roles of UCPs may be manifested at stages of tumorigenesis with genomic instability, where elevated ROS of activated redox signaling are required for further strengthening of malignancy (377). This is consequently prevented by functional activation of, for example, UCP2, the expression of which is frequently elevated in cancer. In accordance with the preventive role, the UCP2 ablation predisposed mice for enhanced tumorigenesis in the proximal colon (98). A mild UCP3-mediated uncoupling has been shown to link catabolism of lipids to inhibition of Akt kinase signaling and relevant resistance in tumorigenesis (304). Thus UCP3 overexpression acted as prevention of aerobic glycolysis suppressing Akt activation in primary human keratinocytes (304).

Antioxidant protection has been indicated in melanoma mediated by UCP2 under umbrella of mitochondrial biogenesis regulation by PGC-1α and upstream melanocortin 1 receptor and BRAF (397). Involvement of UCP2 within information signaling and redox regulations during tumorigenesis involving hypoxia has been recently exemplified by demonstration that upon hypoxic UCP2 downregulation, a chemoresistance is promoted (423). Sensitization of pancreas cancer to glycolysis inhibition has also been reported due to the antioxidant role of UCP2 (46).

Similar results were reported for breast cancer cells (332). Also estrogens may increase mitochondrial superoxide formation by repressing UCP2 in papillary thyroid cancer cells (163). Prooxidative state established by genipin blockage of UCP2 retarded pancreatic adenocarcinoma cell growth and, in contrast, UCP2 overexpression diminished basal autophagy (89). Similarly, the prooxidative state was set up by glutathionylation catalyst diamine selectively deactivating UCP2 in leukemia Mx2 cells. This led to sensitization of drug-resistant leukemia cells to chemotherapeutics, indicating that manipulation of UCP2 glutathionylation status can serve as a therapeutic strategy for cancer treatment (326).

### I. Involvement of UCPs in immune cells

Increasing evidence in the past decade has indicated the crucial role of UCPs in redox signaling in various types of immune cells (Fig. 17). Retrograde redox signaling from mitochondria to the cytoplasm of immune cell activates pathways such as NFκB and HIF, besides the nuclear factor of activated T cells (282). Retrograde redox signaling affects the immune cell proliferation (354), participates in altering their “decision” and differentiation into another cell type, and in apoptosis initiation. UCP2 has also been found to promote engulfment of apoptotic cells by increasing the phagocytic capacity (245). Moreover, a significant UCP2 role in inflammation, by uncoupling-promoted IL-1α production, has been described (126).

UCP2 was shown to be expressed in macrophages as well as in dendritic cells, mastocytes, neutrophils, and also B cells and T cells (17, 349, 389) participating in both innate and adaptive immune responses (119). Even the role of UCPs influencing the leukocyte telomere length has been considered (90). Interestingly, even UCP1 has also been detected in thymocytes (2, 3, 56), where its thermogenic role is probably replaced by a regulatory role in apoptosis due to its ability to attenuate mitochondrial ROS production (104, 106).

Concerning the innate immunity, M1 macrophages produce high levels of proinflammatory cytokines and strongly defeat infection, since they produce large amount of ROS and reactive nitrogen species. Responding to LPS and IFNγ, M1 macrophages are activated to induce nitric oxide synthase and cytokines IL-1β and TNF. In contrast, M2 macrophages, involved in tissue remodeling and tumor growth promotion, possess anti-inflammatory function. Both types can regulate a switch from OXPHOS to aerobic glycolysis and *vice versa*, by which their function is controlled. Various types of redox signaling are involved in their physiology. Active UCPs might attenuate or block such redox signaling. At first, the retrograde redox signaling from mitochondria is required for toll-like receptor (TLR)-stimulated inflammatory cytokine production (428). The source of superoxide has not been established, specifically for LPS stimulation, nevertheless, reverse electron transport to complex I may be involved (283).

In macrophages, UCP2 is expressed as a response to oxidative stress (141). UCP2 negatively controls mitochondrial ROS production in M1 macrophages and this effect is independent of NOX (22, 302, 424). In M1 macrophages, a controlled blockage of UCP2 regulates the strength of oxidative burst (17, 22). The lack of UCP2-mediated antioxidant and anti-inflammatory protection (*i.e*., the lack of attenuation of mitochondria-released superoxide) in a synergy with elevated ROS-perpetuated MAPK signaling (118) results in increased macrophage ability to defeat infection (17, 349). We may also deduce that besides promotion of redox signaling, the antioxidative capacity within mitochondria and cell is decreased at the UCP blockage, since it must deal with the enhanced superoxide formation, and this allows a higher strength for the classic M1 macrophage-activated NOX superoxide formation. Moreover, UCP2 downregulation/inactivation leads to proinflammatory interleukins, again due to redox signaling originating probably from complex I or III (282).

The involvement of UCP2 in TLR4-induced ROS signaling was evidenced in primary cultures of macrophages. Upon stimulation by LPS, UCP2 is quickly downregulated by JNK and p38 pathways (Fig. 17). The consequence is an enhanced mitochondrial ROS production that stimulates both p53 and ERK pathways as a positive feedback signal (118). Thus, decrease of UCP2 levels is a required phenomenon to promote mitochondrial ROS-dependent MAPK signaling during macrophage activation (119). During this process, cellular use of glucose is increased while glutamine oxidation is maintained (81, 409). At the same time, FA utilization is decreased and directed away from mitochondrial oxidation (159). This is in accordance with the assumption that UCP2 downregulation favors mitochondrial glucose oxidation, which enhances mitochondrial ROS production and potentiates MAPK activation (118).

In proinflammatory M1 macrophages that accumulate in adipose tissue during obesity-linked metabolic diseases, UCP2 expression is blocked by adipocyte FA binding protein (FABP4/aP2), and the resulting release of redox signaling is involved in inflammasome activation and IL-1β secretion (161, 385). Inflammasomes are cytoplasmic, multiprotein complexes involved in the sensing of danger signals, the role of which is to trigger caspase-1 activation and insufficient interleukin IL-1β maturation in response to diverse *stimuli*, sensed by pattern recognition receptors such as RIG-I-like receptors (sensing viral RNA), mitochondrial antiviral signaling protein, and TLR9 (sensing mtDNA). In this way, inflammasomes act in host defense against microbial infection. Also NOD-, LRR-, and pyrin domain-containing protein 3 (NLRP3) is an important sensor acting in response to mitochondrial ROS, hence to retrograde redox signaling. As reported, the influence of UCP2 on this has to be clarified as various interrelationships between expression of either one, UCP2 or NLRP3 (340). Autophagy is allowed with activated UCP2 and is inhibited with UCP2 blockage (442). Such autophagy inhibition was explained by the redox signaling ongoing at switched off UCP2 (442).

UCP2 expression is induced in antigen-stimulated CD8^+^ T cells where it is involved in metabolic reprogramming, leading to the differentiation and clonal expansion of the T cells (63). Binding of an antigen to the T cell receptor of naive T cells leads to a metabolic shift from catabolic (FA oxidation) to anabolic state. The burst of ROS is required for T cells activation (367). The redox state needs to be strictly regulated for the optimal clonal expansion of the functionally competent CD8^+^ T cells and also generation of the memory T cells. Expression of UCP2 correlates with stimulation of the T cells by antigen. The UCP2 affects Δ*Ψ*_m_ and thus attenuates mitochondrial superoxide formation in these cells (63). The active UCP2 in this way downregulates the redox signaling required for proinflammatory cytokines in both CD8^+^ T cell and CD4^+^ T cell subsets (414).

Involvement of UCPs in neurodegenerations such as multiple sclerosis has also been investigated. During the development of autoimmune encephalomyelitis, UCP2 is upregulated in spinal cord and this correlates with activation of proper T-lymphocytes (381). Thus, T cell UCP2 is upregulated during neuroinflammation.

## IX. Future Prospects

Mitochondrial retrograde redox signaling as a part of numerous physiological processes should be studied in detail. Even for the best and already verified examples, detailed mechanisms should be investigated for switching on and switching off the redox signals (which reciprocally applies to UCP function). Challenges of the future research also lie in the possible UCP involvement in the transformations of *pro*-OXPHOS type of metabolism to OXPHOS dormancy and in extension into the cancer-specific types of metabolism.

Mechanistically, further studies are required for UCP synergy with phospholipases, nicotinamide nucleotide transhydrogenase, isocitrate dehydrogenase-2, protein machinery of fusion and fission (and neuron retrograde/antiretrograde mitochondrial movement), other SLC25 family transport carriers, key enzymes of specific metabolic modes, key elements of redox and information signaling, and key elements of immune processes in immune cells.

## Abbreviations Used

Δ*p*: protonmotive force
Δ*Ψ*_m_: mitochondrial inner membrane potential
Akt: protein kinase B
ANT: ADP/ATP carrier, or adenosine nucleotide translocase
AQP: aquaporin
C11: undecanesulfonate
C18: octadecanesulfonate
ECM: extracellular matrix
EMRE: essential mitochondrial calcium uniporter regulator
FA(s): fatty acid(s)
FCCP: carbonyl cyanide 4-(trifluoromethoxy)phenylhydrazone
FOXA1: forkhead box A1
GDP: guanosine diphosphate
GPR40: G-protein-coupled receptor-40
GSH: glutathione
GSIS: glucose-stimulated insulin secretion
HIF1-α: hypoxia-inducible factor 1-α
HNE: 4-hydroxyl-2-nonenal
hnRNP K: heterogeneous nuclear ribonucleoprotein K
I/R: ischemia/reperfusion
ICS: intracristal space
IMM: inner mitochondrial membrane
IPC: ischemic preconditioning
iPLA_2_γ: calcium-independent phospholipase A2γ
MAPK: mitogen-activated protein kinase
MCU: mitochondrial calcium uniporter
MICU1: methylation of mitochondrial Ca^2+^ uptake 1
MnSOD: superoxide dismutase 2
NAD^+^: nicotinamide adenine dinucleotide
NLRP3: NOD-, LRR-, and pyrin domain-containing protein 3
NOX: NADPH oxidase
OAA: oxaloacetate
OleA: oleic acid
OMM: outer mitochondrial membrane
OXPHOS: oxidative phosphorylation
PGC-1α: PPARγ coactivator1-α
PHD: proline hydroxylase domain
PKC: protein kinase C
PPARs: peroxisome proliferator-activated receptors
PRMT1: protein arginine methyl transferase 1
ROS: reactive oxygen species
Sp1: specific protein-1
SREBP: sterol regulatory element binding protein
SREs: sterol regulatory elements
TLR: toll-like receptor
TREs: thyroid hormone response elements
TXNIP: thioredoxin-interacting protein
UCP: uncoupling protein
UCP3 KO: UCP3 knockout
UTR: untranslated region
wt: wild-type
